# Putative Causal Variants Are Enriched in Annotated Functional Regions From Six Bovine Tissues

**DOI:** 10.3389/fgene.2021.664379

**Published:** 2021-06-23

**Authors:** Claire P. Prowse-Wilkins, Jianghui Wang, Ruidong Xiang, Josie B. Garner, Michael E. Goddard, Amanda J. Chamberlain

**Affiliations:** ^1^Faculty of Veterinary and Agricultural Science, The University of Melbourne, Parkville, VIC, Australia; ^2^Agriculture Victoria, AgriBio, Centre for AgriBiosciences, Bundoora, VIC, Australia; ^3^Agriculture Victoria, Ellinbank Dairy Centre, Ellinbank, VIC, Australia

**Keywords:** bovine, ChIP-seq, histone modifications, function, causal variants, differential binding, annotation, ChromHMM

## Abstract

Genetic variants which affect complex traits (causal variants) are thought to be found in functional regions of the genome. Identifying causal variants would be useful for predicting complex trait phenotypes in dairy cows, however, functional regions are poorly annotated in the bovine genome. Functional regions can be identified on a genome-wide scale by assaying for post-translational modifications to histone proteins (histone modifications) and proteins interacting with the genome (e.g., transcription factors) using a method called Chromatin immunoprecipitation followed by sequencing (ChIP-seq). In this study ChIP-seq was performed to find functional regions in the bovine genome by assaying for four histone modifications (H3K4Me1, H3K4Me3, H3K27ac, and H3K27Me3) and one transcription factor (CTCF) in 6 tissues (heart, kidney, liver, lung, mammary and spleen) from 2 to 3 lactating dairy cows. Eighty-six ChIP-seq samples were generated in this study, identifying millions of functional regions in the bovine genome. Combinations of histone modifications and CTCF were found using ChromHMM and annotated by comparing with active and inactive genes across the genome. Functional marks differed between tissues highlighting areas which might be particularly important to tissue-specific regulation. Supporting the cis-regulatory role of functional regions, the read counts in some ChIP peaks correlated with nearby gene expression. The functional regions identified in this study were enriched for putative causal variants as seen in other species. Interestingly, regions which correlated with gene expression were particularly enriched for potential causal variants. This supports the hypothesis that complex traits are regulated by variants that alter gene expression. This study provides one of the largest ChIP-seq annotation resources in cattle including, for the first time, in the mammary gland of lactating cows. By linking regulatory regions to expression QTL and trait QTL we demonstrate a new strategy for identifying causal variants in cattle.

## Introduction

Finding the genetic variants which lead to different phenotypes has been the goal of geneticists for many years. Bridging the genotype to phenotype “gap” has linked genes to their functions and identified causes of disease. In the dairy industry, finding genetic variants which affect phenotypes would improve selective breeding using genomic selection (MacLeod et al., 2016). Genomic selection relies on associations between genotypes and phenotypes to predict the phenotypes of animals. But this association could be based on linkage disequilibrium (LD) between a SNP and the causal variant rather than a direct effect of the SNP itself. Therefore, identifying the causal variant would prevent breakdown of LD over time and extend genomic predictions to populations with different LD (Hayes et al., 2016).

Most traits of interest to the dairy industry are complex traits which are predicted to have many causal variants of small effect (Goddard et al., 2016). Because the individual effect of each causal variant is small, these are difficult to find using classical genetics methods, especially in a large long-lived mammal. Studies in humans and other species have shown that trait-associated variants from genome wide association studies (GWAS) are enriched in functional regions of the genome, such as regulatory or protein coding regions (Maurano et al., 2012; Schaub et al., 2012; Trynka et al., 2013; Ma et al., 2015). Apart from genes, most functional regions are not well annotated in the bovine genome which has hampered attempts to ask the same question in cattle (Koufariotis et al., 2014). However, two recent studies found that functional regions identified in cattle were more likely to contain QTL than other regions (Wang et al., 2017; Fang et al., 2019). This reveals the exciting proposition that if functional regions can be identified in the bovine genome, this can narrow down the space in which we search for causal variants.

While genes can be broadly identified using sequence homology, other types of functional elements do not have easily identifiable features, lack sequence conservation and can be located far from genes (Kellis et al., 2014). One study, using homology with human regulatory regions, indicated that these regions in cattle were enriched for QTL (Nguyen et al., 2018), however, recent evidence showed that the use of functional annotations predicted from humans in cattle is limited (Xiang et al., 2019; Raymond et al., 2020). This suggests that identifying functional regions directly in cattle is optimal. Accordingly, the FAANG consortium for the Functional Annotation of ANimal Genomes has been set up to jointly annotate functional regions in livestock genomes by assaying directly for them in relevant species and tissues (Andersson et al., 2015).

Some functional regions are marked by histone modifications – post translational modifications to the histone proteins which DNA is wrapped around in the cell (Zhou et al., 2011). For example, the histone protein H3 has a tail which can be modified by mono (H3K4Me1) or tri-methylation (H3K4Me3) at its 4^*th*^ lysine (Kimura, 2013). Numerous studies (Bernstein et al., 2005; Roh et al., 2006; Heintzman et al., 2007) have found that H3K4Me3 is found at promoters of genes, with one study (Guenther et al., 2007) showing that up to 75% of genes in human embryonic stem cells were marked by H3K4Me3 at their promoter. Other studies have found that H3K4Me1 also marks promoters (Barski et al., 2007; Robertson et al., 2008) and that the regulatory DNA sequences called enhancers, which enhance the transcription of genes (Pennacchio et al., 2013), are marked by H3K4Me1 and sometimes H3K4Me3 (Barski et al., 2007; Robertson et al., 2008; Spicuglia and Vanhille, 2012). The tail of histone H3 can also be modified by acetylation (H3K27ac) or tri-methylation (H3K27Me3) at its 27^*th*^ lysine (Kimura, 2013). Studies have found that active genes and enhancers tended to be marked by H3K27ac (Creyghton et al., 2010) while repressed regions were marked by H3K27Me3 (Zhao et al., 2007; Tie et al., 2009). Potential functional regions can also be marked by other factors. The zinc finger protein CTCF (CCCTC-binding factor) has many functions in the genome. CTCF acts as a transcription factor which can block and activate gene expression, an insulator by blocking interactions between enhancers and promoters, and is involved in the machinery that regulates chromatin conformation (Ong and Corces, 2014; Kim et al., 2015). Assaying the location of these five marks should identify the location of many functional regions in the bovine genome.

The locations of histone modifications and transcription factors can be assayed across the genome using Chromatin Immunoprecipitation followed by sequencing (ChIP-seq) (Park, 2009). Chromatin is fixed so that DNA is bound to the proteins it is interacting with and antibodies are used to isolate the protein of interest, such as a histone modification or transcription factor. The DNA bound to these proteins is then sequenced and aligned to the genome with reads forming “peaks” at the location where the protein was bound. These peaks can be used to annotate putative functional regions in the genome singularly, or by combining data from several proteins (Park, 2009; Ernst and Kellis, 2010). The height of the peaks (characterised by read counts) is also useful. Peak height has been used to predict the expression of nearby genes (Karlić et al., 2010) and variants which associate with peak height have been shown to overlap with variants associated with gene expression (McVicker et al., 2013).

**FIGURE 1:**
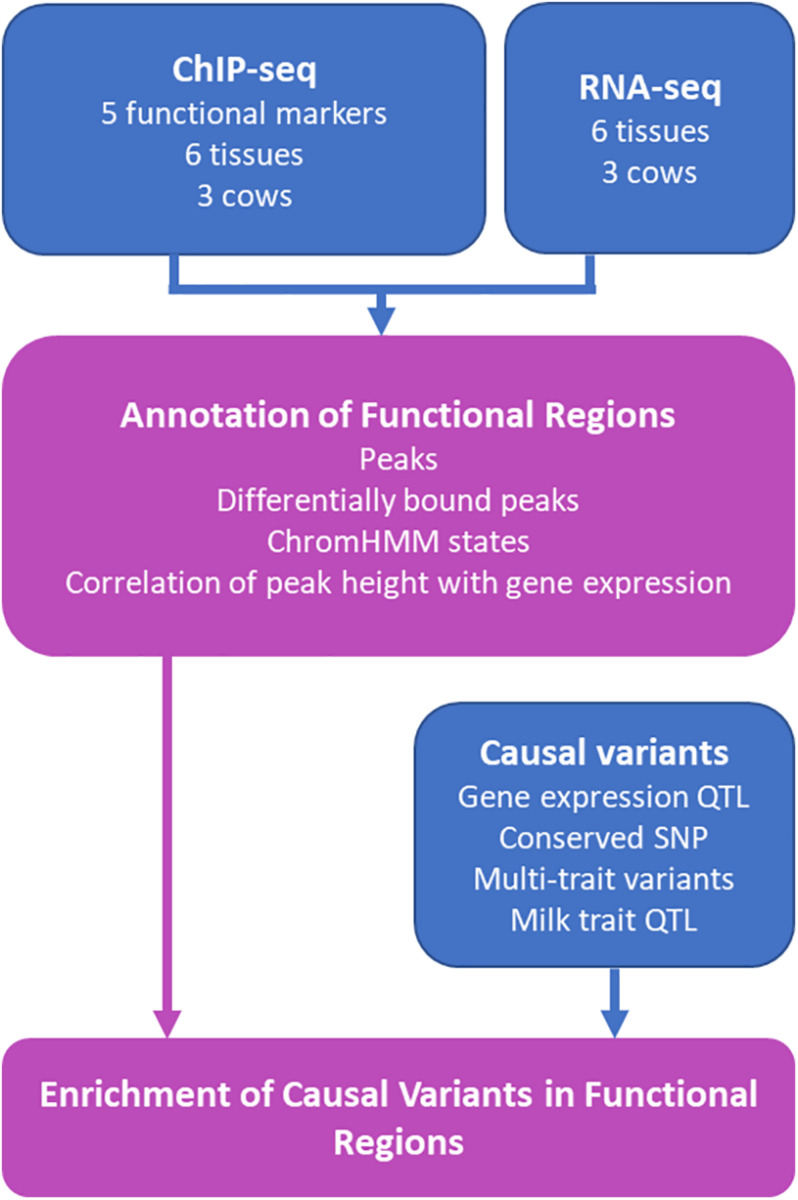
Schematic of Study. Results from the study are outlined in purple boxes while data sources contributing to the results are represented in blue. RNA-seq and ChIP-seq data was generated from heart, liver, lung, kidney, mammary gland, and spleen from the same three cows. The five functional markers assayed were H3K4Me1, H3K4Me3, H3K27Me3, H3K27ac, and CTCF. Details of causal variants are outlined in Table 1.

Functional regions have been identified in the bovine genome in liver (Villar et al., 2015), rumen epithelial cells (Fang et al., 2019) and other tissues (Kern et al., 2021). However, functional regions can vary between tissues (Kellis et al., 2014). This study aimed to increase the catalog of functional regions in the bovine genome by using ChIP-seq to assay the genomic locations of one transcription factor and 4 histone modifications in 6 tissues in 2–3 lactating Holstein dairy cows (Figure 1). This data was used to annotate putative functional regions in the bovine genome and identify tissue specific functional regions. We showed that peak height in some regions correlated with the level of gene expression in nearby genes. Lastly, we confirm that QTL and eQTL are enriched in these putative functional regions.

## Materials and Methods

### Chromatin Immunoprecipitation and RNA Sequencing

Tissue from liver, lung, mammary gland, kidney, heart, and spleen from three lactating Holstein dairy cows were sampled after euthanasia (Chamberlain et al., 2015; Dorji et al., 2020). Ethics approval for the euthanasia and sampling of two of the cows were obtained from Department of Jobs, Precincts and Regions Ethics Committee (Application No. 2014-23). These animals were euthanised out of line of sight of other animals and sedated with 600mg of xylazine IV and 300mg of ketamine before 1l of 25% magnesium sulphate was injected intravenously until animal was deceased. The Third cow was not euthanised for the purposes of this study but because she injured her leg, for this reason the local Animal Ethics Commitee (DEPI Agricultural Research and Extension Animal Ethics Commitee) advised ethics approval was not required. This animal was euthanised by captive bolt.

Tissues were dissected, snap frozen in liquid nitrogen and stored at −80°C until use. Frozen tissue was ground for 3 min in the Geno/Grinder (SPEX SamplePrep). Ground, frozen tissue was fixed for 10 min with 10% formaldehyde and chromatin prepared using the Magnify Chromatin Immunoprecipitation kit (ThermoFisher) as per the manufacturer’s instructions. Fixed chromatin was sheared to 200–500 bp using the Covaris S2 (Covaris). Mammary and liver chromatin was sheared for 3 min, duty cycle five, %intensity four and 200 cycles per burst 200. The remaining tissue were sheared at duty cycle two, %intensity three, 200 cycles per burst for 5–15 min.

Chromatin immunoprecipitation was performed using the Magnify Chromatin immunoprecipitation kit (ThermoFisher) with some modifications. For the mammary and liver each sample was immunoprecipitated in three separate reactions with 10 ul of chromatin and 0.25 ug or 0.5 ug of antibody for the histone modifications and 10 μl of antibody for CTCF. Triplicate samples were combined after de-crosslinking using MinElute PCR purification kit (QIAGEN). For spleen, heart, kidney and lung, 1.5–10 μg chromatin was used for immunoprecipitation with 0.5–0.15 μg antibody (H3K4Me1, H3K4Me3, H3K27ac, and H3K27Me3) per reaction or 10 μl of CTCF antibody. The DNA obtained from ChIP was purified and concentrated using Monarch Genomic DNA Purification Kit (New England Biolabs).

Sequence libraries for ChIP and a corresponding input sample were prepared with the NEBNext Ultra II DNA Library Prep Kit for Illumina (New England Biolabs) each with unique barcodes as per the manufacturer’s instructions and run on the HiSeq 3000 (Illumina) in a 150 cycle paired end run.

Each library was sequenced to between 20 and 300 million reads. Raw sequence reads were trimmed of adapters and poor-quality ends using Trimmomatic (Bolger et al., 2014) removing base pairs from the 3′ and 5′ ends of the sequence if their quality was less than 20 and excluding trimmed reads with length less than 50 bp. Trimmed reads were mapped to the bosTau8/UMD-3.1.1 bovine genome with BWA mem using default settings (Li, 2013). Poor quality reads were removed with Samtools (Li et al., 2009) using *q* > 15 and duplicate reads removed. ChIP and input reads were used to call peaks with MACS2 default settings (Zhang et al., 2008). Quality checks of peaks was performed with deepTools plotFingerprint (Ramírez et al., 2016) and SPP (Kharchenko et al., 2008).

RNA extraction and sequencing on the same six tissues in the three cows was as described in Chamberlain et al. (2015) and Dorji et al. (2020).

### Profile Plots and Replicate Comparisons

Plots for the profile of ChIP-seq reads for each mark were generated using deepTools (Ramírez et al., 2016). First bigWig files were created from mapped ChIP and input reads using the command bamCompare with bin size 10 and using the RPKM option (Reads Per Kilobase of transcript, per Million mapped reads) to normalise the number of reads between samples. Output bigWig scores were the log_2_ ratio of ChIP to input. The command computeMatrix was used to calculate scores at each mark around transcription start sites (TSS) taken from Ensembl [Release 94 (Hunt et al., 2018)]. Active and inactive TSS were determined using the RNAseq data described in Chamberlain et al. (2015). Active TSS were defined when all samples had a count more than 200 at that gene and inactive when all animals had a count less than 10. Matrices were visualised using the command plotProfile.

Similarity between samples was also calculated using deepTools plotCorrelation (Ramírez et al., 2016). The command multiBigWigSummary was used to summarise bigwig files across each mark in bins of 100 bp. The command plotCorrelation was used to generate a heatmap of these values using Pearson correlations.

### Differential Binding Between Tissues

Differential binding (DB) between tissues was calculated using edgeR (Robinson et al., 2010) for consensus peaks. To define a set of consensus peaks for each mark each position in the genome was defined as under a peak or not. Positions which were under a peak in two or more samples were included in the consensus peak dataset. Reads were normalised using edgeR and DB tested by defining a design matrix for which the intercept was the mean in all tissues. Peaks were considered significantly differentially bound with a *P* value less than 0.05 and binding greater than two-fold different to the average binding of that peak in all other tissues.

### Correlation Between Peak Height and Gene Expression Level

The correlation between consensus peak counts and gene count for every peak within 100 kb of a gene TSS across 16 samples (4 tissues X 3 cows and 2 tissues X 2 cows) for H3K27Me3 and H3K27ac or 18 samples (6 tissues X 3 cows) for the other marks was calculated.

Counts for each set of consensus peaks (described above) were calculated with DiffBind (Stark and Brown, 2011) and normalised using Trimmed Mean of M-values (TMM) and full library size. Normalised RNA-seq counts for each gene were used from Chamberlain et al. (2015) and Dorji et al. (2020). Correlations were calculated using corr.test in R.

### Annotation With ChromHMM

Chromatin states were defined using ChromHMM (Ernst and Kellis, 2012). Filtered and deduplicated input and ChIP-seq bam files were binarized using the binarizeBam option with default settings. Between five and 24 states were learned using the LearnModel function of ChromHMM. A model with 7 states was chosen for further analysis as in this model each state was unique.

To annotate, chromatin states were compared to known regions in the genome using the OverlapEnrichment function in ChromHMM. Locations of TSS and genes were downloaded from Ensembl [release 94 (Hunt et al., 2018)]. The promoter region was characterised as 2kb upstream of the TSS and proximal regions as 8 kb upstream from the promoter start site. Normalised read counts from RNA-seq data (Chamberlain et al., 2015; Dorji et al., 2020) were used to define genes, and their associated promoters and proximal regions, as active or inactive. Active genes were defined when all animals had a count over 200 in that tissue and for inactive genes all animals had a count less than 10 in that tissue. We also looked for tissue-specific active genes, these were when all animals had counts above 200 in that tissue and below 10 in all other tissues.

### Enrichment of Putative Causal SNPs in Functional Regions

Enrichment of putative causal SNPs in peaks, peaks which correlated with gene expression, differentially bound peaks and ChromHMM states was calculated. Enrichment was calculated using the formula outlined in ChromHMM (Ernst and Kellis, 2012) and described below.

Enrichment = (C/A)/(B/D) where: A is the number of positions under peaks, B is the number of positions that were putative causal SNPs, C is the number of positions under peaks and also a putative causal SNP and D is the number of positions in the genome.

The significance of enrichment or depletion was calculated using a hypergeometric test in R. A variety of putative causal SNP datasets were used for enrichment analysis (Table 1). There were no SNP on the X chromosome in the datasets so only autosomal regions were tested. Total genome size was calculated as the sum of chromosomes 1–29.

**TABLE 1:** Putative causal SNPs.

Dataset	Number of SNPs	Description	References
Allele specific eQTL	1,100,446	Allele specific expression QTL from white blood cells and milk cells in 112 holstein cows (*P* < 0.0001)	Chamberlain et al. (2018)
Exon eQTL	945,832	Exon expression QTL from white blood cells, milk cells, liver, and muscle in 209 holstein cows (*P* < 0.0001)	Xiang et al. (2018); Xiang et al. (2019)
Gene eQTL	110,200	Gene expression QTL from white blood cells, milk cells, liver, and muscle in 209 holstein cows (*P* < 0.0001)	Xiang et al. (2018); Xiang et al. (2019)
Conserved regions	378,472	SNPs conserved in 100 species lifted over from human to bovine genome	Xiang et al. (2019)
SNP 80k	83,454	Top 80,000 sequence variants ranked for their contributions to 34 traits	Xiang et al. (2019); Xiang et al. (2021)
Splice QTL	1,112,324	Splice QTL from blood, milk cells, liver, and muscle in 209 holstein cows (*P* < 0.0001)	Xiang et al. (2018); Xiang et al. (2019)
Protein Yield QTL	3,317	QTL from GWAS in 32,347 cows for protein yield with *P* < 1 × 10^–7^	Xiang et al. (2020)
Fat yield QTL	4,815	QTL from GWAS in 32,347 cows for fat yield with *P* < 1 × 10^–7^	Xiang et al. (2020)
Milk Yield QTL	6,883	QTL from GWAS in 32,347 cows for milk yield with *P* < 1 × 10^–7^	Xiang et al. (2020)
Fat percentage QTL	12,373	QTL from GWAS in 32,347 cows for fat percentage with *P* < 1 × 10^–7^	Xiang et al. (2020)
Protein percentage QTL	17,012	QTL from GWAS in 32,347 cows for protein percentage with *P* < 1 × 10^–7^	Xiang et al. (2020)

*Details of each SNP dataset used in enrichment analysis.*

### Enrichment Based on Location in the Genome

We tested whether enrichment of putative causal SNPs within peaks changed depending on location in the genome. Enrichment was calculated with only peaks and SNPs located within 100 kb from a TSS, 100–200 kb from a TSS and so on up to a million base pairs from a transcription start site. Transcription start sites for each gene were taken from Ensembl [Release 94 (Hunt et al., 2018)]. The distance to the nearest TSS for each peak was calculated from the summit (as defined by MACS2) of narrow peaks and the midpoint of broad peaks (halfway between the start and end of the peak).

### Filtering SNPs for Linkage Disequilibrium

To account for SNPs in high linkage disequilibrium (LD), the GWAS SNP datasets were filtered for LD using plink (Purcell et al., 2007). Within each 1mb window, with a moving step of 50 SNPs, SNPs were retained if they had pairwise *r*^2^ < 0.5. Enrichment was calculated with these filtered SNP datasets as described above.

## Results

### Description of ChIP-seq Peaks

86 ChIP-seq datasets were generated as shown in Supplementary Table 1.

Quality of the ChIP-seq assay was assessed by calculating the Jensen-Shannon Distance (JSD) between the sample and input and cross strand correlation metrics. All but two samples exceeded ENCODE standards for cross correlation metrics (NSC > 1.05 and RSC > 0.9, Supplementary Table 2). However, we found JSD to be a more reliable metric, which was less sensitive to read depth. All ChIP-seq data had a JSD between 0.23 and 0.5 (Supplementary Table 2).

After filtering of poor-quality reads and removal of duplicates, between 24,847,326 and 316,216,350 mapped ChIP-seq reads remained for each sample (Supplementary Table 2). To account for bias in shearing, library preparation and mapping, an input control using the same batch of sheared chromatin was also sequenced for each sample. Mapped ChIP-seq and input control bam files were used in MACS2 (Zhang et al., 2008) to call between 31,303 and 871,452 peaks for each sample. The average size of peaks was 400 bp to 600 bp for peaks designated as “narrow” in MACS2 (H3K27ac, H3K4Me3, and CTCF) and 1000 bp to 1100 bp for peaks called as “broad” (H3K4Me1 and H3K27Me3, Table 2). An average of 13% of the genome was under a peak in any one dataset. When considering narrow peaks, the percentage of genome covered in each dataset was strongly correlated with number of mapped reads (*r* = 0.715, *P* < 0.001) but was less strongly correlated in broad peaks (*r* = 0.363, *P* = 0.036). Number of peaks per dataset was also strongly correlated with the number of mapped reads (*r* = 0.656, *P* < 0.001) (Supplementary Figure 1).

**TABLE 2:** Summary of peaks.

Mark	Average number of peaks	Average mapped reads	Average% of genome	Average size of peaks
H3K4Me3	419,386	132,285,209	7	560
H3K4Me1	513,830	136,545,806	21	1,163
H3K27ac	493,905	154,628,688	10	583
H3K27Me3	555,459	127,247,856	21	1,049
CTCF	456,881	90,288,075	7	410

*For each mark, the average number of peaks, mapped reads, percentage of genome under peaks, and average size of peaks are reported.*

### Profile Plots and Replicate Comparisons

**FIGURE 2:**
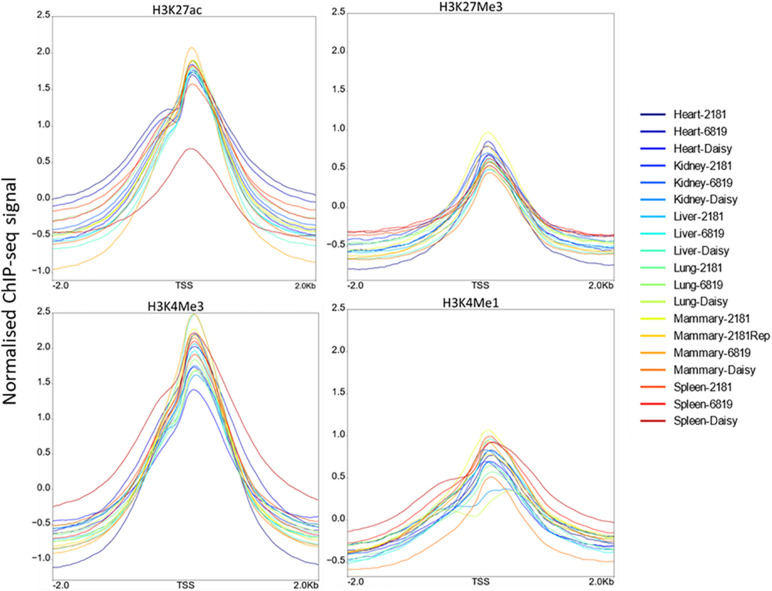
Distribution of ChIP-seq reads around the transcription start site (TSS). For each histone modification these plots show the normalised ChIP-seq signal above input signal within 2 kb of the TSS.

Profile plots display the normalised ChIP-seq signal above input signal. As expected H3K4Me3 displayed the highest signal around the TSS, followed by H3K27ac. The profiles for H3K4Me3, H3K4Me1, and H3K27ac displayed a slight bimodal shape at the TSS (Figure 2). When comparing the profiles around the TSS of 475 active genes and 8,398 inactive genes, H3K27ac samples showed high signal near the TSS of active genes while H3K27Me3 samples showed lower signal (Supplementary Figure 2).

We compared replicates within marks using Pearson correlations. In general, all samples were strongly correlated regardless of tissue or animal, however, there were some batch effects (Supplementary Figure 3).

### Differential Binding Between Tissues

Normalised ChIP-seq counts at consensus peaks in each tissue were compared for differential binding. Peaks were defined as differentially bound where the mean of the counts in one tissue was significantly different (*P* < 0.05) and two-fold higher or lower than the mean of the counts in all other tissues.

The largest number of differentially bound peaks were in H3K27ac where almost 24% of peaks were different between tissues (Table 3). Of the H3K27ac DB peaks, a large proportion were higher in heart (Supplementary Figure 4).

**TABLE 3:** Differentially bound peaks.

Mark	Peaks tested	Peaks DB	Percentage peaks DB
H3K27ac	885,919	213,293	24.1
H3K4Me3	853,788	30,965	3.6
H3K4Me1	649,485	21,230	3.3
H3K27Me3	740,188	17,347	2.3
CTCF	961,881	93,473	9.7

*Number of peaks tested as well as number and percentage of peaks differentially bound (DB) between tissues (*P* < 0.05, fold change > 2).*

### Correlation Between Peak Height and Gene Expression Level

**FIGURE 3:**
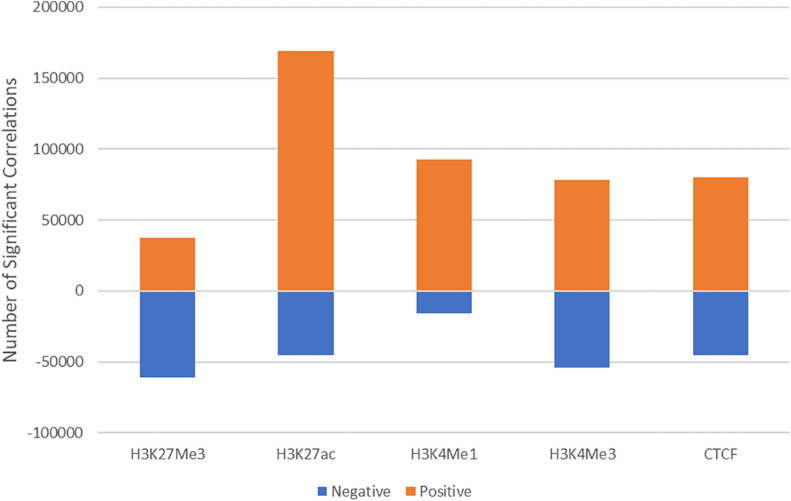
Direction of significant correlations. The direction of correlations between ChIP-seq and RNA-seq counts for H3K27Me3, H3K27ac, H3K4Me1, H3K4Me3, and CTCF.

We examined whether variation in peak height correlated with variation in gene expression. Normalised ChIP-seq counts at consensus peaks in each mark were compared to normalised gene counts from RNA-seq in the same sample. Every peak within 100 kb of a gene was tested for an association between peak height and gene expression. This resulted in more than 1 million tests for each mark. It was not expected that there would be a correlation between all these peak-gene pairs, however, it was unknown which peaks were interacting with which genes, so it was necessary to test them all. Although the sample size was low (16 or 18 depending on the mark), there was a significant (*P* < 0.05) correlation in 6–11% of peak-gene pairs, which is more than the 5% expected by chance. Similarly, most correlations were negative for H3K27Me3 and positive for all other marks which would not be expected if the correlations were random chance (Figure 3).

### Annotation With ChromHMM

Using the four histone modifications and CTCF seven chromatin states were defined across the genome (Table 4) using ChromHMM (47). When higher numbers of states were tested ChromHMM defined states with similar functional mark profiles at similar probabilities. To annotate the seven states, we looked for enrichment within these states within active and inactive genes, promoters and proximal regions and used evidence from other studies of what these marks represent. Between 1,169 and 1,826 genes were defined as active in any one tissue and between 10,653 and 11,815 defined as inactive. There were between six and 134 genes which were only active in one tissue (Supplementary Table 4).

**TABLE 4:** Emission probabilities for seven states from ChromHMM.

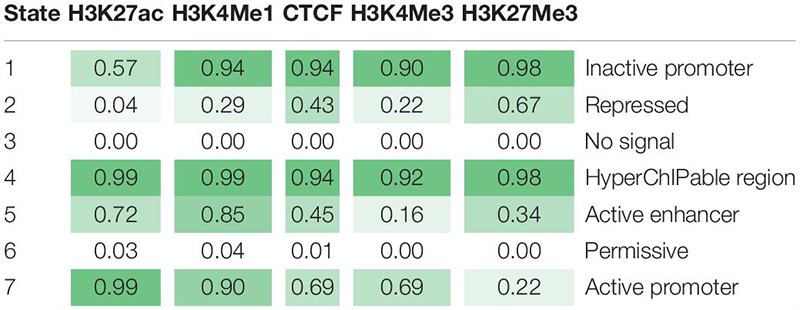

*The emission probabilities for each mark in each state provided by ChromHMM. Darker colour indicates higher probability. Annotations (last column) for each state was based on location in the genome and known associations from literature.*

**FIGURE 4:**
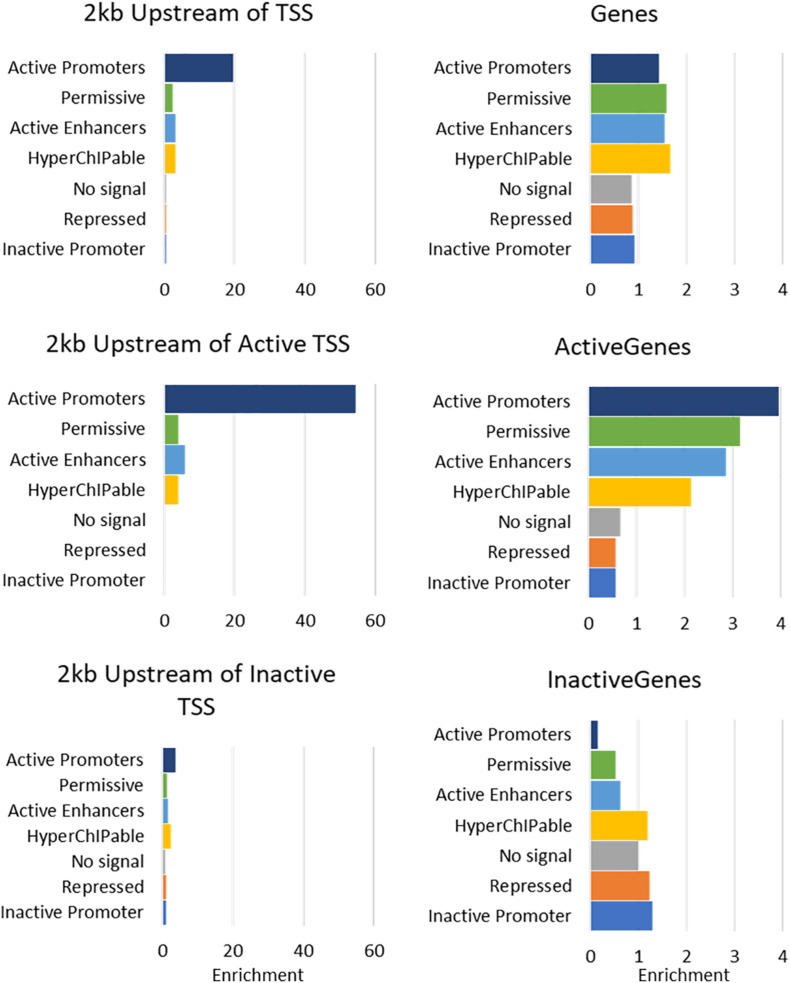
Enrichment of 7 Chromatin states in Mammary Gland at annotated regions of the bovine genome. For more details of enrichment at annotated regions see Supplementary Figure 5.

The seven states were annotated as inactive promoters, repressed, no signal, hyperChIPable region, active enhancer, permissive, and active promoter (Table 4). State 2 was annotated as “repressed” as it had a high chance of observing the inactive mark H3K27Me3 and was enriched in inactive genes (Figure 4). State 1 was defined as “inactive promoter” because it was slightly more enriched upstream of inactive genes (except for in liver) consistent with inactive promoters and had a high probability of observing H3K27Me3 but also H3K4Me1, H3K4Me3, and CTCF. Two states (3 and 6) displayed low probability of any mark. State 3 was defined as “no signal” as it covered most of the genome but was not highly enriched in any annotated regions suggesting regions which were not functional at that time in these tissues. State 6 was annotated as “permissive” as it was highly enriched in active genes and regions proximal to active genes indicating a more open, permissive state. State 5 was termed “active enhancer” as it had a high probability of H3K27ac and H3K4Me1 (Table 4) which is a combination thought to denote active enhancers (26). This state was slightly enriched in active genes in all tissues (Figure 4). State 7 had a high probability of observing H3K4Me1, H3K4Me3, CTCF, and the activating mark H3K27ac (Table 4) and was defined as “active promoter.” This state was enriched at all regions 2 kb upstream of the TSS but was particularly enriched upstream of active TSS (Figure 4). State 4 had a high probability of observing all 5 marks at once and was enriched in both active and inactive regions but did not show a consistent pattern in any of the regions tested. We defined State 4 as “hyperChIPable regions” as coined in a recent study (Massa et al., 2021).

Analysis of tissue specific genes was hampered by the small numbers involved (Supplementary Table 4); Kidney, lung and spleen only had 14, 11, and six active genes identified as tissue specific. However, in mammary and liver (26 and 134 tissue specific genes, respectively) active tissue specific genes, promoters, and proximal regions were enriched in “active promoters” similar to other active genes, promoters, and proximal regions (Supplementary Table 5). However, in heart, tissue specific active genes, promoters and proximal regions were more enriched for “hyperChIPable regions” than “active promoters” which was not consistent with the other active genes, promoters, and proximal regions.

**FIGURE 5:**
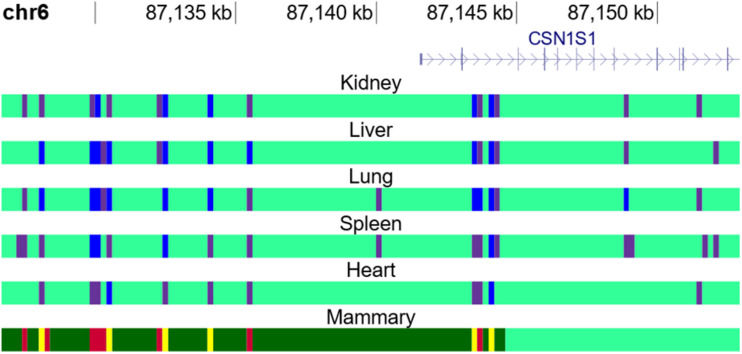
ChromHMM states vary between tissues. Representation of ChromHMM states in heart, kidney, liver, lung, spleen, and mammary gland tissue near the transcription start site of CSN1, a gene highly expressed in the mammary gland. “Inactive promoter” (blue), “Repressed” (purple) and “No Signal” (light green) states are all inactive states, while “Active Enhancer” (red), “HyperChIPable Region” (yellow), and “Permissive” (dark green) are all active states.

Inspection of ChromHMM states near known genes showed clear delineation between active and inactive genes in appropriate tissues (e.g., Figure 5). Alpha S1 casein (*CSN1*) is a gene known to be highly expressed in the mammary gland (Ibeagha-Awemu et al., 2016). The gene expression data showed that it is highly expressed in mammary tissue but not in the other tissues studied here. Figure 5 shows mammary tissue is marked by hyperChIpable (yellow), active enhancer (red) and permissive (dark green) states, all active states, while the remaining tissues are marked by inactive promoter (blue), repressed (purple), and no signal (light green) states, all inactive states. State 7 is not present, which is meant to be an active promoter state.

### Enrichment of Putative Causal SNPs in Functional Regions

Putative functional regions described above were tested for enrichment of potential causative SNPs from 11 datasets that included expression QTL, milk production trait QTL and sites conserved across numerous species (see materials and methods Table 1 for descriptions of the datasets).

#### Enrichment in Peaks

**FIGURE 6:**
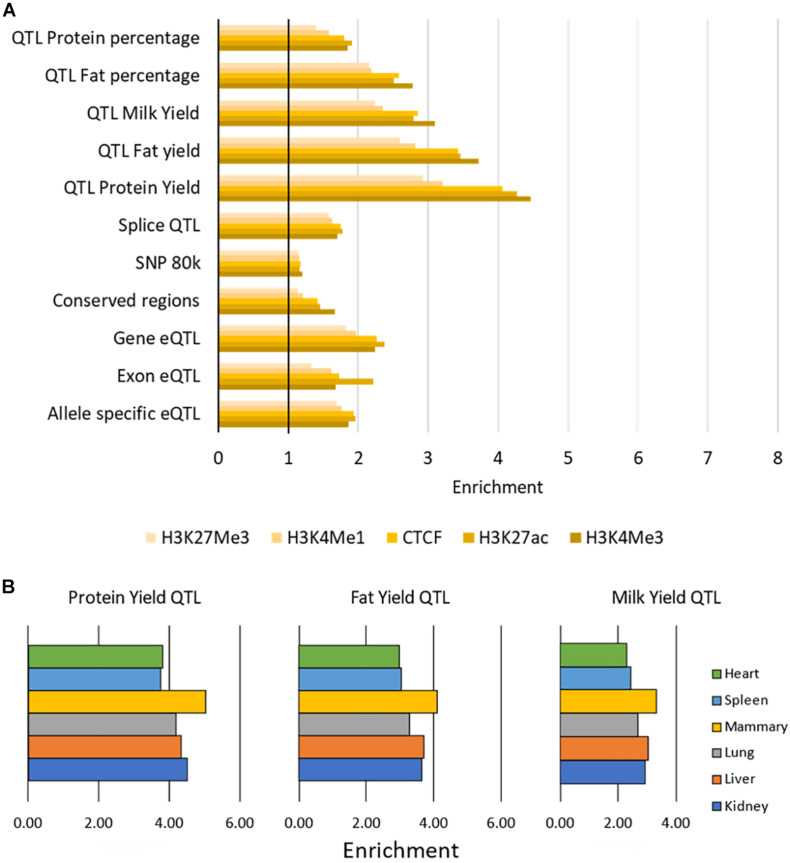
Enrichment of causal variants within functional regions. **(A)** Enrichment of causal variants within peaks. Enrichment of each SNP dataset within each histone modification or CTCF averaged across tissues. All values above 1 (indicated by the vertical black bar) are enriched for causal variants. Enrichment was significant with *P* < 0.001 for all tests. **(B)** Enrichment of three sets of milk trait QTL within H3K27ac peaks. Peaks in mammary gland have the highest enrichment for these milk trait QTL.

All putative causal variants were significantly overrepresented in peaks (enrichment > 1) with average enrichment between 1.17 (for SNP80k) and 3.82 (for QTL Protein Yield) for each SNP dataset (Figure 6 and Supplementary Table 6). The narrow peaks (H3K4Me3, H3K27ac, and CTCF) had the highest enrichment across all SNP datasets although the differences were small. The QTL for protein yield had the highest enrichment across all marks.

There was little variation in enrichment between tissues, except in the milk production QTL results where H3K4Me3, H3K27ac, and CTCF peaks in mammary gland consistently had higher enrichment (e.g., Figure 6, Supplementary Table 6). All enrichment was highly significant with *P* < 0.001 (Supplementary Table 6).

#### Enrichment in Differentially Bound Peaks

Some differentially bound peaks were enriched for causal variants and some were depleted (Supplementary Table 7). This was not consistent across marks or across tissues. This likely reflects the small numbers of peaks which were differentially bound in some cases (Table 3) which increased the noise in the data.

#### Enrichment in Peaks Which Correlate With Gene Expression

**FIGURE 7:**
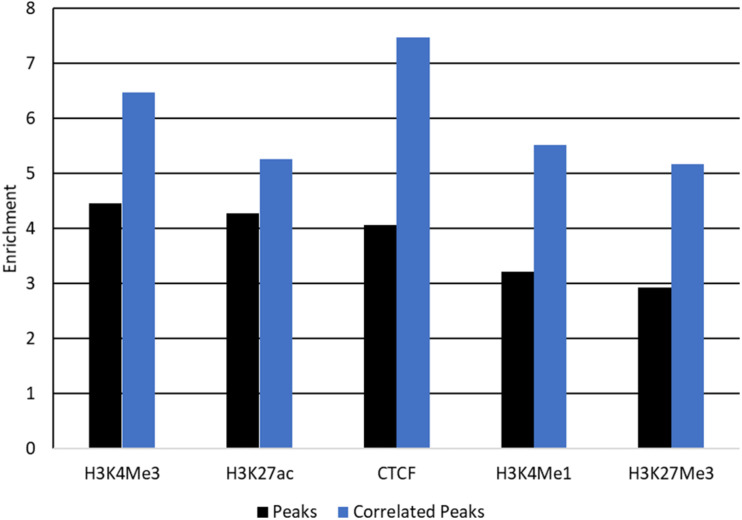
Enrichment of QTL for protein yield in peaks correlated with gene expression. Enrichment of QTL for protein yield within peaks (black) and peaks correlated with gene expression (blue) for each mark. Enrichment was significant with *P* < 0.001 for all tests.

Peaks which correlate with gene expression may be affecting gene expression and so are strong candidate regions for causal variants. Filtering peaks for those correlated with gene expression (Supplementary Table 8) improved enrichment for all SNP sets except SNP 80 k and conserved regions (Figure 7 and Supplementary Table 9). Enrichment was significant in all cases with *P* < 0.001.

#### Enrichment in ChromHMM States

**FIGURE 8:**
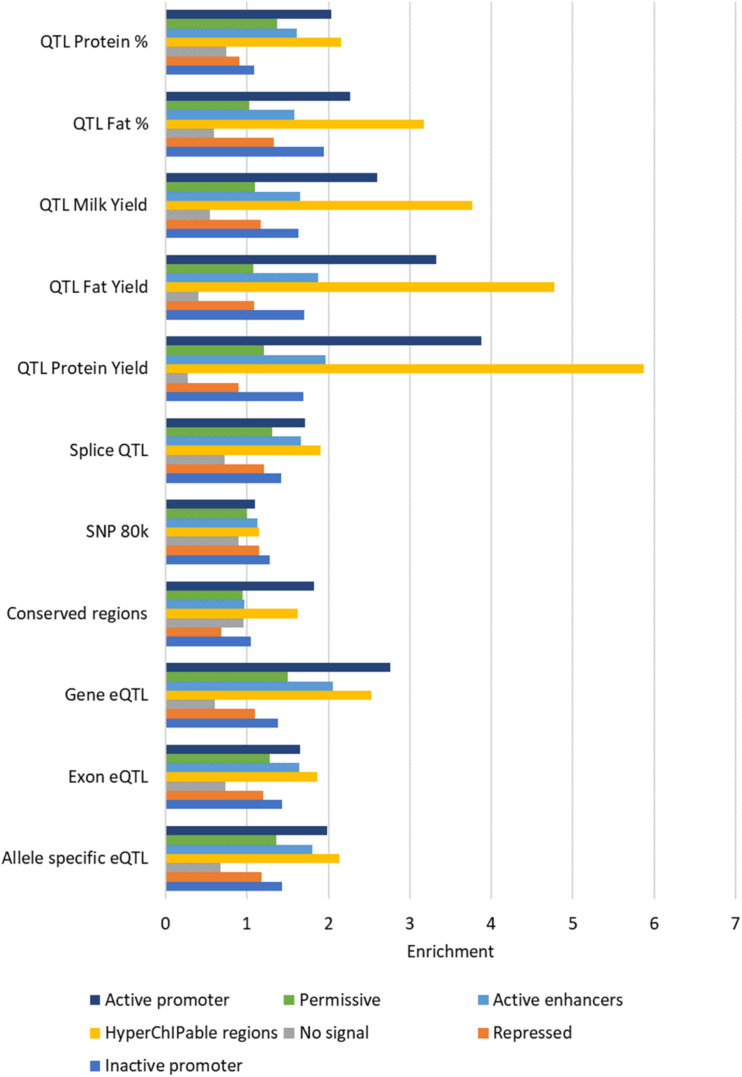
Average enrichment of chromatin states for each SNP dataset. Enrichment for each state averaged across tissues.

All the states defined by ChromHMM except state 3 (“no signal”) were enriched for some causal SNP datasets (Figure 8). The states with the highest enrichment were State 4 (“hyperChIPable region”) and State 7 (“active promoters”) except for the 80 k SNPs which were most enriched in State 1 (“inactive promoters”). The highest enrichment was for QTL for protein yield in State 4 (“hyperChIPable region”), these QTL also had the lowest enrichment in State 3 (“no signal”), where they were strongly depleted. Most of the SNP datasets showed highest enrichment in states 4 and 7, moderate enrichment in states 1,2,5, and 6 and depletion in state 3. However, the 80 k SNP dataset showed low to no enrichment in all states except State 1 and conserved SNPs showed low to no enrichment in all states except 4 and 7. The highest enrichment in State 4 was only slighter better than the highest enrichment when considering peak regions defined by the histone mark and was worse than when using peaks correlated with gene expression. Depletion and enrichment were statistically significant in most cases (Supplementary Table 10).

Consistent with results in peaks there was little variation in enrichment between tissues in most of the SNP datasets except for conserved regions and milk production QTL (Supplementary Table 10). Conserved regions were largely similar between tissues except for heart which had higher enrichment in State 1 than the other tissues. State 1 in heart was also consistently more enriched in all five milk production QTL SNP datasets than the other tissues. State 7 in liver and in some cases mammary displayed little to no enrichment for the milk production QTL while the other tissues displayed high enrichment for these QTL in this state.

### Confounding Factors

#### Enrichment Based on Location in the Genome

One confounding factor of the enrichment analysis is that potential causal SNP may be found within peaks because both peaks and causal SNP may tend to be found close to genes. We tested whether enrichment of potential causal SNPs in peaks changed depending on the distance of the peaks from the nearest gene and whether this explained the high enrichment seen within peaks. Peaks and SNPs were split up into 10 groups of 100 kb increments (Supplementary Tables 11, 12) depending on their distance to the nearest TSS and enrichment was tested within these groups.

**FIGURE 9:**
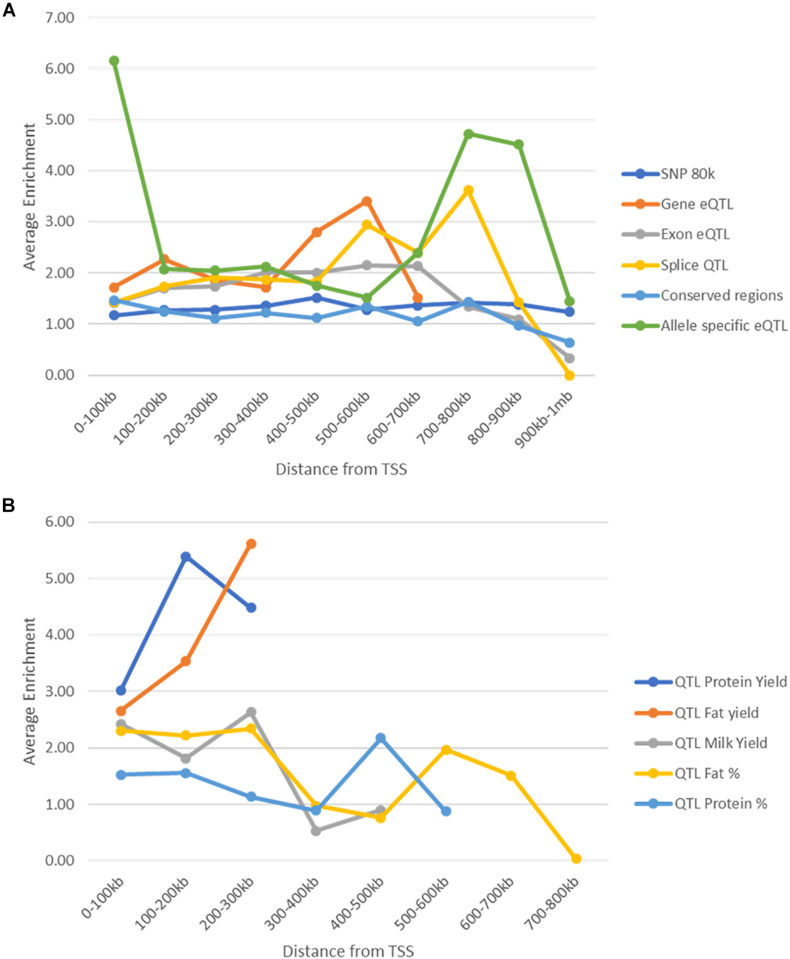
Enrichment of SNPs within peaks by distance to the nearest transcription start site. **(A)** Enrichment (averaged across all animals, tissues, and marks) of SNPs within peaks at different distances from transcription start sites. Enrichment stays above 1 in all cases until 800 kb from the nearest transcription start site. **(B)** Enrichment (averaged over all tissues and animals) of milk production QTL within peaks at different distances from transcription start site. Enrichment was not calculated for protein and fat yield more than 300 kb from a TSS as there were less than 10 SNPs.

As distance from TSS increased the number of SNPs and peaks in these regions decreased (Supplementary Tables 11, 12). In general enrichment of most of the SNP datasets within peaks remained constant regardless of location (Figure 9). However, conserved SNPs were not enriched in any tissues or marks more than 100 kb from TSS and Exon eQTL and Splice QTL were not enriched further than 800 kb from TSS. Allele specific eQTL remained enriched within peaks in all regions but the strongest enrichment was between 0 and 100 kb from TSS (Figure 9). Milk production QTL SNPs were not found more than 300 kb from a TSS (Supplementary Table 11) so enrichment could not be assessed beyond this, but peaks were enriched for SNPs up to this point (Figure 9).

#### Filtering SNPs

**FIGURE 10:**
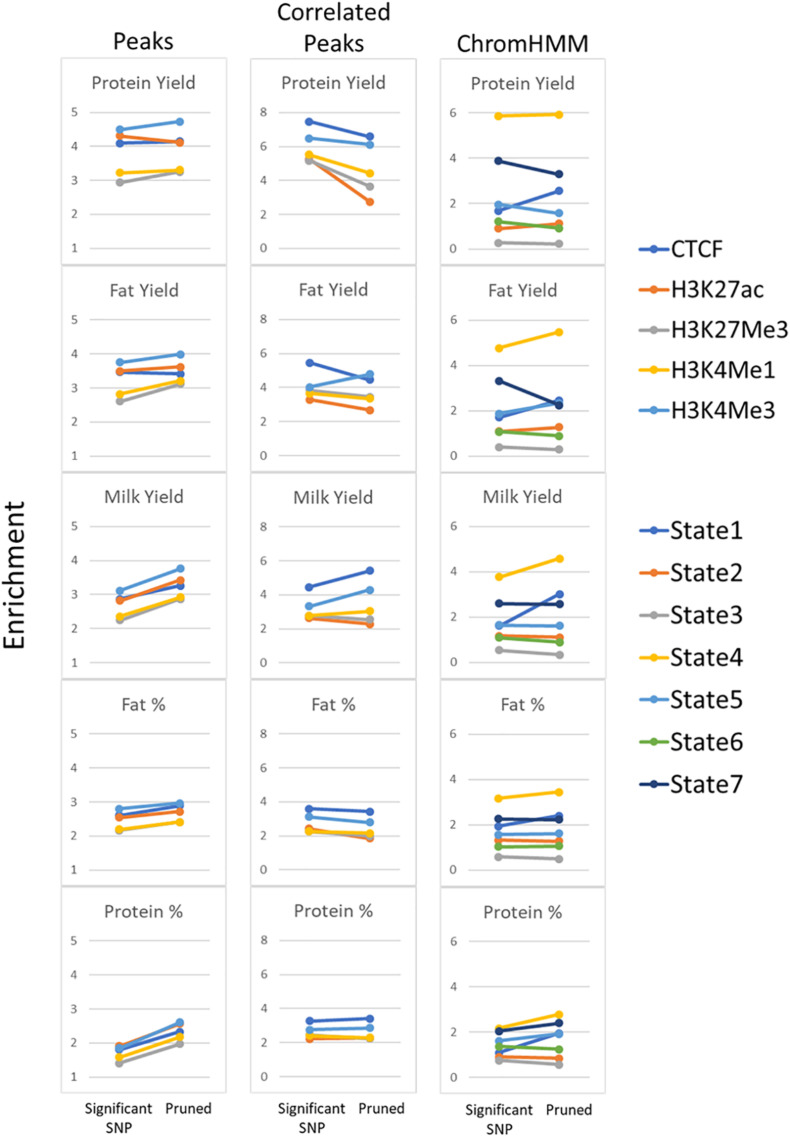
Enrichment of pruned SNPs within functional regions. Comparison of enrichment for all significant milk production QTL (*P* < 1 × 10^–7^) compared with pruning SNPs at *r*^2^ < 0.5. All peaks and peaks correlated with gene expression (Correlated Peaks) for all 5 traits are shown as well as ChromHMM states.

Another confounding factor of this analysis is that high enrichment might be driven by a group of SNPs all in high LD with each other. To avoid this possibility significant milk production QTL SNPs (*P* < 1 × 10^–7^) were filtered to account for linkage disequilibrium (LD pruning) where SNPs were retained with r^2^ < 0.5 (Supplementary Table 13). Pruning improved enrichment in peaks but not in peaks whose height was correlated with gene expression (Figure 10). Pruning consistently improved enrichment in State 1 and 4 but had mixed effects in other states.

## Discussion

This study presents the results of ChIP-seq for 4 histone modifications and one transcription factor in six tissues from 3 lactating dairy cows. The ChIP-seq data was used to annotate functional regions in the bovine genome and establish whether functional regions are enriched for causal variants.

To confirm the quality of the ChIP-seq data we looked at the intensity of ChIP signal for each histone modification within 2 kb of the TSS (Figure 2). All 4 histone modifications had an increase at the TSS. H3K4Me3 had the highest intensity consistent with expectations (Guenther et al., 2007) followed by H3K27ac. Some studies show a bimodal distribution of H3K4Me1 and/or H3K4Me3 signal at promoters (Barski et al., 2007; Kingsley et al., 2020) but this is not always observed (Hoffman et al., 2010; Bae and Lesch, 2020). In this study only a very slight bimodal distribution in the profile of H3K4Me3, H3K4Me1, and H3K27ac was observed. We also plotted the profile of the active and inactive marks H3K27ac and H3K27Me3 at active and inactive genes (Supplementary Figure 2). H3K27ac had much higher signal than H3K27Me3 at active genes as expected (Barski et al., 2007) but there was only a small difference between active and inactive gene profiles in H3K27Me3. This modest difference has also been found in other studies (Barski et al., 2007) with Mikkelsen et al. (2007) finding that not all repressed promoters are marked by H3K27Me3.

For each functional mark, peaks were called from ChIP DNA sequence using MACS2. Up to 500,000 peaks were found for each mark representing millions of functional regions in the genome of dairy cows. Differentially bound peaks were also annotated. Only very low numbers of peaks were different between tissues in most of the marks except for H3K27ac (Table 3). This is consistent with high correlation among all samples as observed in Supplementary Figure 3. It is not surprising that H3K27ac differed between tissues more than other marks because it is thought to be associated with active promoters and enhancers (Barski et al., 2007). However, work in other species suggests that the enhancer associated mark H3K4Me1 should also display some tissue specificity while marks such as H3K4Me3 are more uniform (Heintzman et al., 2009; Shen et al., 2012). This study did not find this. There were notable differences in the number of differentially bound peaks between tissues. For example, 24% of the H3K27ac regions tested were different in at least one tissue but more than half of these differentiated regions were specific to heart (Supplementary Figure 4). In addition, CTCF in mammary and liver had extremely high numbers of down-regulated peaks and very few up-regulated peaks, while kidney, lung and spleen had the opposite. The addition of more samples in future will likely improve this result.

To annotate peaks which are correlated with gene expression, all peaks within 100 kb of a transcription start site were tested for a correlation between read counts under the peak and in the gene. As expected, most peak-gene pairs were not correlated, however, there were more significant correlations than would be expected by chance. For most marks, a high proportion of correlations were positive which would also not occur by chance. The highest number of correlated peaks were from histone modification H3K27ac (Figure 3), this is logical as this mark has been found to correlate with active regions of the genome (Wang et al., 2008; Cotney et al., 2012). Similarly, the majority of H3K27Me3 correlations were negative (Figure 3), which is also expected as this mark has been found to represent repression of transcriptional activity (Barski et al., 2007; Cotney et al., 2012). These peaks are important functional regions to annotate as they indicate a potential functional link between significantly correlated peaks and nearby gene expression.

ChromHMM (Ernst and Kellis, 2012) was used to combine data from multiple marks and call seven chromatin states across the genome. RNA-seq data from the same tissues were used to annotate these states. The results show a demarcation between active and inactive states consistent across multiple tissues. For example, ChromHMM was able to differentiate State 3 (“no signal”), which covered most of the genome, from State 6 (“permissive”), which was highly enriched at active genes, even though they had very similar histone modification and transcription factor occupancy. However, this was not always consistent. State 7 (“active promoters”) was highly enriched at active promoters but there was also some enrichment at inactive promoters. Similarly State 2 (“repressive”) was depleted in active regions but was only slightly enriched in inactive regions. Lastly, State 1 which we called “Inactive promoters” was only slightly enriched upstream of inactive genes. Genes were defined as inactive when there was no RNA-seq counts from that gene which is very stringent, so it is possible some of these genes were poised or were not accurately annotated.

The remaining states were annotated based on information from other studies. State 5 was labelled an “active enhancer” because the marks present in this state reflect enhancer conditions in other species (Heintzman et al., 2007; Creyghton et al., 2010), however, we were unable to provide any genomic evidence for this as enhancers are poorly annotated in the bovine genome. State 4 (“hyperChIPable regions”) had all five marks occurring in the same place and was found to varying degrees in all genomic regions looked at. This phenomenon was described in a recent study and was termed a “hyperChIPable” region (Massa et al., 2021). However, this is not consistent with most other published literature which suggests H3K27ac and H3K27Me3, active and inactive marks, respectively, should not occur at the same place in the genome (Tie et al., 2009). We hypothesise this could occur for multiple, not mutually exclusive reasons. (1) These regions are “poised” between active and inactive states in the mass of heterogeneous cells in the tissue. (2) One or more of the peaks are false positives found when the antibody binds off-target (Jain et al., 2015). (3) High sequence depth is picking up weak peaks which are not found at “normal” read depths (20 million reads are recommended by ENCODE (Landt et al., 2012), our data is between 20 and 300 million reads). (4) These regions represent an important functional region. It seems likely that all 4 options are contributing to our observations including the fact that some of these peaks are false positives. Despite this we found these regions were highly enriched for putative causal SNPs which suggests some function.

It is hypothesised that causal variants are found in functional regions of the genome. To test whether this is true for this study we tested multiple potential causal variant datasets for enrichment in the functional regions described in this study. The enrichment of ChIP-seq peaks for potential causal variants in all datasets was significantly higher than random, although the degree varied across datasets, tissues, and marks. ChIP-seq peaks in mammary gland were particularly enriched for SNPs identified in GWAS for milk production traits. This is consistent with other studies showing trait-associated variants are enriched in peaks from tissues associated with the trait (Ernst et al., 2011; Kundaje et al., 2015). The ChromHMM state with a high probability of all five marks occurring together (State 4-“hyperChIPable regions”) was also enriched for putative causal SNPs while the state with no signal from any functional marks was depleted for all SNP datasets tested. The highest enrichment was observed in peaks which correlated with gene expression. Except for SNPs from conserved regions and the 80 k SNPs, all SNP datasets were highly enriched in these peaks. A potential causal mechanism for these variants would be that they affect the functional mark binding which in turn affects gene expression making these good candidate SNPs for further investigation.

A limitation of the enrichment study is that none of the SNPs tested were confirmed causal variants. Most were SNPs which associate with a phenotype so may be in linkage disequilibrium with a causal variant but are not the causal variant themselves. This means that the enrichment of causal variants (the proportion of which will be different in different datasets) will be diluted by the non-causal variants in the dataset, but it could also mean that our functional regions were just enriched for SNPs in linkage disequilibrium with causal variants. However, the data shows that across multiple SNP datasets using different methodologies there was consistent enrichment for these SNPs within functional regions. This is consistent with studies in human and cattle (Maurano et al., 2012; Schaub et al., 2012; Trynka et al., 2013; Ma et al., 2015; Wang et al., 2017; Fang et al., 2019).

Another possible limitation is that the peaks were enriched because they and the SNPs tested were both near genes. This would mean they are more likely to intersect because of similar distributions in the genome rather than due to a causal variant affecting functional mark binding (Cano-Gamez and Trynka, 2020). To test this, we split the genome into regions based on how far each was from the nearest TSS and tested enrichment with peaks and SNPs just within these regions. In most cases SNPs were enriched in ChIP-seq peaks regardless of the distance of the region being tested from a TSS. This suggests that the enrichment observed was not just a function of proximity to genes. Unfortunately, this could only be tested up to 300 kb in the milk trait GWAS dataset as there were too few SNPs more than 300 kb from a TSS. Due to the small number of SNPs in the GWAS datasets and the fact that they mostly cluster together we were concerned that multiple SNPs from few locations were enriched in few peaks and these SNPs were all tagging one causal variant. To account for this, SNPs were filtered for LD and only the most significant was included from each LD region. This reduced the number of SNPs dramatically but in most cases filtering in this way either improved enrichment or did not change it suggesting successful pruning of SNPs which were in LD with the one causal variant and peaks were still enriched for these variants.

**FIGURE 11:**
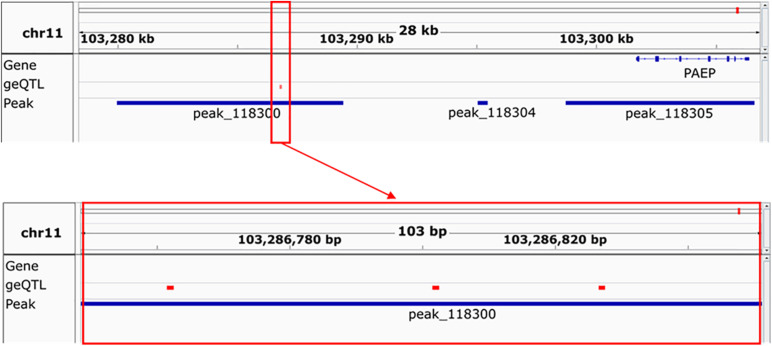
An example of the utility of this data. Peak 118,300, whose location is shown in the upper panel, is significantly 2-fold higher in mammary gland than other tissues and its height (not shown) correlates (*r* = 0.71, *P* = 0.002) with the expression of the gene PAEP. Three geQTL, shown in the red box in the upper and lower panels, which correlate with the expression of PAEP in milk cells, can be found in this peak.

To highlight the utility of the data generated, an example of three SNPs which we hypothesise are good candidate causal variants for important milk traits is shown (Figure 11). In a gene expression QTL study in milk cells, Xiang et al. (2020) found 531 SNPs which significantly associate with expression of the progestagen-associated endometrial protein gene *PAEP* (Ibeagha-Awemu et al., 2016). In this study, we filtered these 531 SNPs down to three (rs208362116, rs210272536, and rs136737193) that were found in a H3K27ac peak which was higher in mammary gland than the other five tissues. The height of this peak also correlated with *PAEP* gene expression (*r* = 0.71, *P* = 0.002). A genetic variant in this peak which altered its height therefore may also alter *PAEP* gene expression. Further study would be needed to verify this, but ChIP-seq data enabled us to filter 531 SNPs down to three genetic variants with a potential causal mechanism for altering gene expression.

Genomic selection will be more accurate over time and across breeds if causal variants are included in predictive modelling (MacLeod et al., 2016). It is thought that causal variants are found in functional regions of the genome but until recently (Villar et al., 2015; Fang et al., 2019; Kern et al., 2021) these were not well annotated in cattle (Koufariotis et al., 2014; Nguyen et al., 2018). This study annotated functional regions in six tissues in 2–3 Holstein dairy cows using ChIP-seq for four histone modifications and one transcription factor. This is the first time this has been done in the mammary gland of a lactating dairy cow. Although many histone modifications overlapped between tissues, some regions showed a difference in binding across tissues and some peaks were correlated with differences in gene expression. Lastly, we confirmed that putative causal variants were enriched in the functional regions discovered. This confirms that future work should consider using these regions when selecting SNPs for genomic selection (MacLeod et al., 2016; Xiang et al., 2019).

## Data Availability Statement

The sequence data for this study is publicly available in the European Nucleotide Archive at http://www.ebi.ac.uk/ena/data/view/PRJEB41939.

## Ethics Statement

The animal study was reviewed and approved by the Department of Jobs, Precincts, and Regions Ethics Committee (Application No. 2014-23).

## Author Contributions

CP-W contributed to conception and design, acquisition of data, data analysis, and drafting of manuscript. JW contributed to acquisition of data. RX contributed to data analysis and critical revision. JG contributed to data acquisition. MG contributed to conception and design, data analysis, and critical revision. AC contributed to conception and design, data acquisition, data analysis, and critical revision. All authors contributed to the article and approved the submitted version.

## Conflict of Interest

The authors declare that the research was conducted in the absence of any commercial or financial relationships that could be construed as a potential conflict of interest.

## Supplementary Material

Supplementary Figure 1 Pearson correlations of mapped reads with other parameters. **(A)**. The number of peaks found significantly increases as mapped reads increases (*r* = 0.656, *P* < 0.001). **(B)**. The percent of the genome covered by narrow peaks significantly increases as mapped reads increase (*r* = 0.715, *P* < 0.001). **(C)**. The percent of the genome covered by broad peaks significantly increases as mapped reads increase (*r* = 0.363, *P* = 0.036).

Supplementary Figure 2 Comparison of profiles of H3K27Me3 and H3K27ac at active and inactive genes. Normalised ChIP-seq signal above input signal of the active (H3K27ac) and inactive (H3K27Me3) histone modifications within 2kb of transcription start sites (TSS) for active and inactive genes in all six tissues.

Supplementary Figure 3 Comparison of replicates in each mark. Pearson correlations of replicates in each mark.

Supplementary Figure 4 Direction of differential binding. Number of differentially bound peaks with either higher (Up) or lower (Down) binding in heart, liver, kidney, lung, mammary gland (MG), and spleen.

Supplementary Figure 5 Enrichment of 7 Chromatin states in different tissues at annotated regions of the bovine genome. Where state1 is “inactive promoter,” state 2 is “repressed,” state 3 is “no signal,” state 4 is “hyperChIPable,” state 5 is “active enhancer,” state 6 is “permissive,” and state 7 is “active promoter.” Darker blue indicates higher enrichment.

Supplementary Table 1 Summary of 86 ChIP-seq datasets. The number of biological replicates is shown for each tissue-mark combination.

Supplementary Table 2 Summary data for each ChIP-seq library. Information on the number of mapped reads, quality and coverage of the genome for each ChIP-seq dataset.

Supplementary Table 3 Correlation between ChIP-seq and RNA-seq counts. The number of tests for correlation between gene expression and peak height as well as the number and percentage significantly correlated (*P* < 0.05) for each mark. All peaks within 100 kb of a gene were tested.

Supplementary Table 4 Active and inactive genes used for ChromHMM annotation. The number of genes defined as active, tissue-specific active and inactive based on normalised count data from RNA-seq in siz tissues across three animals.

Supplementary Table 5 Enrichment of 7 Chromatin states in different tissues at annotated regions of the bovine genome. Where state1 is “inactive promoter,” state 2 is “repressed,” state 3 is “no signal,” state 4 is “hyperChIPable regions,” state 5 is “active enhancer,” state 6 is “permissive,” and state 7 is “active promoter.” Darker green indicates higher enrichment.

Supplementary Table 6 Enrichment of SNP datasets within peaks. The enrichment of each SNP dataset within peaks for each sample. Significance of enrichment or depletion was determined with a hypergeometric test. A summary table is included to the right with average enrichment for each tissue. Darker green indicates higher enrichment.

Supplementary Table 7 Enrichment of SNP datasets within differentially bound peaks. The enrichment of each SNP dataset within differentially bound peaks for each sample with either higher (Up) or lower (Down) binding. Significance of enrichment or depletion was determined with a hypergeometric test. A summary table is included to the right with average enrichment for each tissue. Darker green indicates higher enrichment.

Supplementary Table 8 Number of peaks correlated with gene expression. Number of peaks for each mark whose height correlated with gene expression.

Supplementary Table 9 Enrichment of putative causal variants of peaks and peaks correlated with gene expression. Enrichment of each SNP dataset within peaks and peaks correlated with gene expression. Filtering for correlation with gene expression increased enrichment for causal variants. Enrichment was significant with *P* < 0.001 for all tests.

Supplementary Table 10 Enrichment of SNP datasets within ChromHMM states. The enrichment of each SNP dataset within each state in each tissue. Significance of enrichment or depletion was determined with a hypergeometric test. A summary table is included to the right with average enrichment for each tissue.

Supplementary Table 11 The number of SNPs in each dataset grouped by distance to nearest transcription start site. For each SNP dataset we grouped SNPs based on distance to nearest transcription start site in bins of 100 kb.

Supplementary Table 12 The number of peaks grouped by distance to transcription start site, averaged over samples. The average number of peaks, grouped by distance to the nearest transcription start site, tested for enrichment.

Supplementary Table 13 Number of SNPs in milk production QTL datasets after filtering using r^2^.

## References

[B1] Andersson L. Archibald A. L. Bottema C. D. Brauning R. Burgess S. C. Burt D. W. (2015). Coordinated international action to accelerate genome-to-phenome with FAANG, the functional annotation of animal genomes project. *Genome Biol.* 16 1–6. 25854118 10.1186/s13059-015-0622-4 PMC4373242

[B2] Bae S. Lesch B. J. (2020). H3K4me1 distribution predicts transcription state and poising at promoters. *Front. Cell Dev. Biol.* 8:289. 10.3389/fcell.2020.00289 32432110 PMC7214686

[B3] Barski A. Cuddapah S. Cui K. Roh T.-Y. Schones D. E. Wang Z. (2007). High-resolution profiling of histone methylations in the human genome. *Cell* 129 823–837. 10.1016/j.cell.2007.05.009 17512414

[B4] Bernstein B. E. Kamal M. Lindblad-Toh K. Bekiranov S. Bailey D. K. Huebert D. J. (2005). Genomic maps and comparative analysis of histone modifications in human and mouse. *Cell* 120 169–181. 10.1016/j.cell.2005.01.001 15680324

[B5] Bolger A. M. Lohse M. Usadel B. (2014). Trimmomatic: a flexible trimmer for Illumina sequence data. *Bioinformatics* 30 2114–2120. 10.1093/bioinformatics/btu170 24695404 PMC4103590

[B6] Cano-Gamez E. Trynka G. (2020). From GWAS to function: using functional genomics to identify the mechanisms underlying complex diseases. *Front. Genet.* 11:424. 10.3389/fgene.2020.00424 32477401 PMC7237642

[B7] Chamberlain A. Hayes B. Xiang R. Jagt C. V. Reich C. MacLeod I. (2018). “Identification of regulatory variation in dairy cattle with RNA sequence data,” in *Proceedings of the World Congress on Genetics Applied to Livestock Production Molecular Genetics*, Vol. 1(Palmerston North: Massey University), 254.

[B8] Chamberlain A. J. Vander Jagt C. J. Hayes B. J. Khansefid M. Marett L. C. Millen C. A. (2015). Extensive variation between tissues in allele specific expression in an outbred mammal. *BMC Genomics* 16:993. 10.1186/s12864-015-2174-0 26596891 PMC4657355

[B9] Cotney J. Leng J. Oh S. DeMare L. E. Reilly S. K. Gerstein M. B. (2012). Chromatin state signatures associated with tissue-specific gene expression and enhancer activity in the embryonic limb. *Genome Res.* 22 1069–1080. 10.1101/gr.129817.111 22421546 PMC3371702

[B10] Creyghton M. P. Cheng A. W. Welstead G. G. Kooistra T. Carey B. W. Steine E. J. (2010). Histone H3K27ac separates active from poised enhancers and predicts developmental state. *Proc. Natl. Acad. Sci. U. S. A.* 107 21931–21936. 10.1073/pnas.1016071107 21106759 PMC3003124

[B11] Dorji J. Vander Jagt C. J. Garner J. B. Marett L. C. Mason B. Reich C. M. (2020). Expression of mitochondrial protein genes encoded by nuclear and mitochondrial genomes correlate with energy metabolism in dairy cattle. *BMC Genomics* 21:720. 10.1186/s12864-020-07018-7 33076826 PMC7574280

[B12] Ernst J. Kellis M. (2010). Discovery and characterization of chromatin states for systematic annotation of the human genome. *Nat. Biotechnol.* 28 817–825. 10.1038/nbt.1662 20657582 PMC2919626

[B13] Ernst J. Kellis M. (2012). ChromHMM: automating chromatin-state discovery and characterization. *Nat. Methods* 9 215–216. 10.1038/nmeth.1906 22373907 PMC3577932

[B14] Ernst J. Kheradpour P. Mikkelsen T. S. Shoresh N. Ward L. D. Epstein C. B. (2011). Mapping and analysis of chromatin state dynamics in nine human cell types. *Nature* 473 43–49. 10.1038/nature09906 21441907 PMC3088773

[B15] Fang L. Liu S. Liu M. Kang X. Lin S. Li B. (2019). Functional annotation of the cattle genome through systematic discovery and characterization of chromatin states and butyrate-induced variations. *BMC Biol.* 17:68. 10.1186/s12915-019-0687-8 31419979 PMC6698049

[B16] Goddard M. Kemper K. MacLeod I. Chamberlain A. Hayes B. (2016). Genetics of complex traits: prediction of phenotype, identification of causal polymorphisms and genetic architecture. *Proc. Royal Soc. B* 283:20160569. 10.1098/rspb.2016.0569 27440663 PMC4971198

[B17] Guenther M. G. Levine S. S. Boyer L. A. Jaenisch R. Young R. A. (2007). A chromatin landmark and transcription initiation at most promoters in human cells. *Cell* 130 77–88. 10.1016/j.cell.2007.05.042 17632057 PMC3200295

[B18] Hayes B. Chamberlain A. Daetwyler H. Vander Jagt C. Goddard M. (2016). Improving genomic selection across breeds and across generations with functional annotation. *J. Anim. Sci.* 94 3–4. 10.2527/jas2016.94supplement43a 32704858

[B19] Heintzman N. D. Hon G. C. Hawkins R. D. Kheradpour P. Stark A. Harp L. F. (2009). Histone modifications at human enhancers reflect global cell-type-specific gene expression. *Nature* 459 108–112. 10.1038/nature07829 19295514 PMC2910248

[B20] Heintzman N. D. Stuart R. K. Hon G. Fu Y. Ching C. W. Hawkins R. D. (2007). Distinct and predictive chromatin signatures of transcriptional promoters and enhancers in the human genome. *Nat. Genet.* 39 311–318. 10.1038/ng1966 17277777

[B21] Hoffman B. G. Robertson G. Zavaglia B. Beach M. Cullum R. Lee S. (2010). Locus co-occupancy, nucleosome positioning, and H3K4me1 regulate the functionality of FOXA2-, HNF4A-, and PDX1-bound loci in islets and liver. *Genome Res.* 20 1037–1051. 10.1101/gr.104356.109 20551221 PMC2909568

[B22] Hunt S. E. McLaren W. Gil L. Thormann A. Schuilenburg H. Sheppard D. (2018). Ensembl variation resources. *Database* 2018:bay119. 10.1093/database/bay119 30576484 PMC6310513

[B23] Ibeagha-Awemu E. M. Li R. Ammah A. A. Dudemaine P.-L. Bissonnette N. Benchaar C. (2016). Transcriptome adaptation of the bovine mammary gland to diets rich in unsaturated fatty acids shows greater impact of linseed oil over safflower oil on gene expression and metabolic pathways. *BMC Genomics* 17:104–104. 10.1186/s12864-016-2423-x 26861594 PMC4748538

[B24] Jain D. Baldi S. Zabel A. Straub T. Becker P. B. (2015). Active promoters give rise to false positive ‘Phantom Peaks’ in ChIP-seq experiments. *Nucleic Acids Res.* 43 6959–6968. 10.1093/nar/gkv637 26117547 PMC4538825

[B25] Karlić R. Chung H.-R. Lasserre J. Vlahovièek K. Vingron M. (2010). Histone modification levels are predictive for gene expression. *Proc. Natl. Acad. Sci. U. S. A.* 107 2926–2931. 10.1073/pnas.0909344107 20133639 PMC2814872

[B26] Kellis M. Wold B. Snyder M. P. Bernstein B. E. Kundaje A. Marinov G. K. (2014). Defining functional DNA elements in the human genome. *Proc. Natl. Acad. Sci. U. S. A.* 111 6131–6138. 24753594 10.1073/pnas.1318948111 PMC4035993

[B27] Kern C. Wang Y. Xu X. Pan Z. Halstead M. Chanthavixay G. (2021). Functional annotations of three domestic animal genomes provide vital resources for comparative and agricultural research. *Nat. Commun.* 12:1821. 10.1038/s41467-021-22100-8 PMC7988148 33758196

[B28] Kharchenko P. V. Tolstorukov M. Y. Park P. (2008). Design and analysis of ChIP-seq experiments for DNA-binding proteins. *Nat. Biotechnol.* 26 1351–1359. 10.1038/nbt.1508 19029915 PMC2597701

[B29] Kim S. Yu N.-K. Kaang B.-K. (2015). CTCF as a multifunctional protein in genome regulation and gene expression. *Exp. Mol. Med.* 47:e166. 10.1038/emm.2015.33 26045254 PMC4491725

[B30] Kimura H. (2013). Histone modifications for human epigenome analysis. *J. Hum. Genet.* 58 439–445. 10.1038/jhg.2013.66 23739122

[B31] Kingsley N. Kern C. Creppe C. Hales E. N. Zhou H. Kalbfleisch T. (2020). Functionally annotating regulatory elements in the equine genome using histone mark chip-seq. *Genes* 11:3. 10.3390/genes11010003 31861495 PMC7017286

[B32] Koufariotis L. Chen Y.-P. P. Bolormaa S. Hayes B. J. (2014). Regulatory and coding genome regions are enriched for trait associated variants in dairy and beef cattle. *BMC Genomics* 15:436. 10.1186/1471-2164-15-436 24903263 PMC4070550

[B33] Kundaje A. Meuleman W. Ernst J. Bilenky M. Yen A. Heravi-Moussavi A. (2015). Integrative analysis of 111 reference human epigenomes. *Nature* 518 317–330. 25693563 10.1038/nature14248 PMC4530010

[B34] Landt S. G. Marinov G. K. Kundaje A. Kheradpour P. Pauli F. Batzoglou S. (2012). ChIP-seq guidelines and practices of the ENCODE and modENCODE consortia. *Genome Res.* 22 1813–1831. 22955991 10.1101/gr.136184.111 PMC3431496

[B35] Li H. (2013). Aligning sequence reads, clone sequences and assembly contigs with BWA-MEM. *arXiv* [Preprint]. https://arxiv.org/abs/1303.3997v2

[B36] Li H. Handsaker B. Wysoker A. Fennell T. Ruan J. Homer N. (2009). The sequence alignment/map format and SAMtools. *Bioinformatics* 25 2078–2079. 10.1093/bioinformatics/btp352 19505943 PMC2723002

[B37] Ma M. Ru Y. Chuang L.-S. Hsu N.-Y. Shi L.-S. Hakenberg J. (2015). Disease-associated variants in different categories of disease located in distinct regulatory elements. *BMC Genomics* 16:S3. 10.1186/1471-2164-16-S8-S3 26110593 PMC4480828

[B38] MacLeod I. Bowman P. Vander Jagt C. Haile-Mariam M. Kemper K. Chamberlain A. (2016). Exploiting biological priors and sequence variants enhances QTL discovery and genomic prediction of complex traits. *BMC Genomics* 17:144. 10.1186/s12864-016-2443-6 26920147 PMC4769584

[B39] Massa A. T. Mousel M. R. Herndon M. K. Herndon D. R. Murdoch B. M. White S. N. (2021). Genome-wide histone modifications and CTCF enrichment predict gene expression in sheep macrophages. *Front. Genet.* 11:612031. 10.3389/fgene.2020.612031 33488675 PMC7817998

[B40] Maurano M. T. Humbert R. Rynes E. Thurman R. E. Haugen E. Wang H. (2012). Systematic localization of common disease-associated variation in regulatory DNA. *Science* 337 1190–1195. 10.1126/science.1222794 22955828 PMC3771521

[B41] McVicker G. van de Geijn B. Degner J. F. Cain C. E. Banovich N. E. Raj A. (2013). Identification of genetic variants that affect histone modifications in human cells. *Science* 342 747–749. 10.1126/science.1242429 24136359 PMC3947669

[B42] Mikkelsen T. S. Ku M. Jaffe D. B. Issac B. Lieberman E. Giannoukos G. (2007). Genome-wide maps of chromatin state in pluripotent and lineage-committed cells. *Nature* 448 553–560. 10.1038/nature06008 17603471 PMC2921165

[B43] Nguyen Q. H. Tellam R. L. Naval-Sanchez M. Porto-Neto L. R. Barendse W. Reverter A. (2018). Mammalian genomic regulatory regions predicted by utilizing human genomics, transcriptomics, and epigenetics data. *GigaScience* 7:gix136. 10.1093/gigascience/gix136 PMC5838836 29618048

[B44] Ong C.-T. Corces V. G. (2014). CTCF: an architectural protein bridging genome topology and function. *Nat. Rev. Genet.* 15 234–246. 10.1038/nrg3663 24614316 PMC4610363

[B45] Park P. J. (2009). ChIP–seq: advantages and challenges of a maturing technology. *Nat. Rev. Genet.* 10 669–680. 10.1038/nrg2641 19736561 PMC3191340

[B46] Pennacchio L. A. Bickmore W. Dean A. Nobrega M. A. Bejerano G. (2013). Enhancers: five essential questions. *Nat. Rev. Genet.* 14 288–295. 10.1038/nrg3458 23503198 PMC4445073

[B47] Purcell S. Neale B. Todd-Brown K. Thomas L. Ferreira M. A. Bender D. (2007). PLINK: a tool set for whole-genome association and population-based linkage analyses. *Am. J. Hum. Genet.* 81 559–575. 10.1086/519795 17701901 PMC1950838

[B48] Ramírez F. Ryan D. P. Grüning B. Bhardwaj V. Kilpert F. Richter A. S. (2016). deepTools2: a next generation web server for deep-sequencing data analysis. *Nucleic Acids Res.* 44 W160–W165. 27079975 10.1093/nar/gkw257 PMC4987876

[B49] Raymond B. Yengo L. Costilla R. Schrooten C. Bouwman A. C. Hayes B. J. (2020). Using prior information from humans to prioritize genes and gene-associated variants for complex traits in livestock. *PLoS Genet.* 16:e1008780. 10.1371/journal.pgen.1008780 32925905 PMC7514049

[B50] Robertson A. G. Bilenky M. Tam A. Zhao Y. Zeng T. Thiessen N. (2008). Genome-wide relationship between histone H3 lysine 4 mono-and tri-methylation and transcription factor binding. *Genome Res.* 18 1906–1917. 10.1101/gr.078519.108 18787082 PMC2593584

[B51] Robinson M. D. McCarthy D. J. Smyth G. K. (2010). edgeR: a Bioconductor package for differential expression analysis of digital gene expression data. *Bioinformatics* 26 139–140. 10.1093/bioinformatics/btp616 19910308 PMC2796818

[B52] Roh T.-Y. Cuddapah S. Cui K. Zhao K. (2006). The genomic landscape of histone modifications in human T cells. *Proc. Natl. Acad. Sci. U. S. A.* 103 15782–15787. 10.1073/pnas.0607617103 17043231 PMC1613230

[B53] Schaub M. A. Boyle A. P. Kundaje A. Batzoglou S. Snyder M. (2012). Linking disease associations with regulatory information in the human genome. *Genome Res.* 22 1748–1759. 22955986 10.1101/gr.136127.111 PMC3431491

[B54] Shen Y. Yue F. McCleary D. F. Ye Z. Edsall L. Kuan S. (2012). A map of the cis-regulatory sequences in the mouse genome. *Nature* 488 116–120. 10.1038/nature11243 22763441 PMC4041622

[B55] Spicuglia S. Vanhille L. (2012). Chromatin signatures of active enhancers. *Nucleus* 3 126–131. 10.4161/nucl.19232 22555596 PMC3383566

[B56] Stark R. Brown G. (2011). *“DiffBind**: Differential Binding Analysis of ChIP-Seq Peak Data”.* Available online at: http://bioconductor.org/packages/release/bioc/vignettes/DiffBind/inst/doc/DiffBind.pdf

[B57] Tie F. Banerjee R. Stratton C. A. Prasad-Sinha J. Stepanik V. Zlobin A. (2009). CBP-mediated acetylation of histone H3 lysine 27 antagonizes Drosophila Polycomb silencing. *Development* 136 3131–3141. 10.1242/dev.037127 19700617 PMC2730368

[B58] Trynka G. Sandor C. Han B. Xu H. Stranger B. E. Liu X. S. (2013). Chromatin marks identify critical cell types for fine mapping complex trait variants. *Nat. Genet.* 45 124–130. 10.1038/ng.2504 23263488 PMC3826950

[B59] Villar D. Berthelot C. Aldridge S. Rayner T. F. Lukk M. Pignatelli M. (2015). Enhancer evolution across 20 mammalian species. *Cell* 160 554–566. 10.1016/j.cell.2015.01.006 25635462 PMC4313353

[B60] Wang M. Hancock T. P. MacLeod I. M. Pryce J. E. Cocks B. G. Hayes B. J. (2017). Putative enhancer sites in the bovine genome are enriched with variants affecting complex traits. *Genet. Sel. Evol.* 49:56. 10.1186/s12711-017-0331-4 PMC5499214 28683716

[B61] Wang Z. Zang C. Rosenfeld J. A. Schones D. E. Barski A. Cuddapah S. (2008). Combinatorial patterns of histone acetylations and methylations in the human genome. *Nat. Genet.* 40 897–903. 10.1038/ng.154 18552846 PMC2769248

[B62] Xiang R. Hayes B. J. Vander Jagt C. J. MacLeod I. M. Khansefid M. Bowman P. J. (2018). Genome variants associated with RNA splicing variations in bovine are extensively shared between tissues. *BMC Genomics* 19:521. 10.1186/s12864-018-4902-8 29973141 PMC6032541

[B63] Xiang R. MacLeod I. M. Daetwyler H. D. de Jong G. O’Connor E. Schrooten C. (2021). Genome-wide fine-mapping identifies pleiotropic and functional variants that predict many traits across global cattle populations. *Nat. Commun.* 12:860. 10.1038/s41467-021-21001-0 33558518 PMC7870883

[B64] Xiang R. van den Berg I. MacLeod I. M. Daetwyler H. D. Goddard M. E. (2020). Effect direction meta-analysis of GWAS identifies extreme, prevalent and shared pleiotropy in a large mammal. *Commun. Biol.* 3:88. 10.1038/s42003-020-0823-6 PMC7048789 32111961

[B65] Xiang R. Van Den Berg I. MacLeod I. M. Hayes B. J. Prowse-Wilkins C. P. Wang M. (2019). Quantifying the contribution of sequence variants with regulatory and evolutionary significance to 34 bovine complex traits. *Proc. Natl. Acad. Sci. U. S. A.* 116 19398–19408. 10.1073/pnas.1904159116 31501319 PMC6765237

[B66] Zhang Y. Liu T. Meyer C. A. Eeckhoute J. Johnson D. S. Bernstein B. E. (2008). Model-based analysis of ChIP-Seq (MACS). *Genome Biol.* 9:R137. 10.1186/gb-2008-9-9-r137 PMC2592715 18798982

[B67] Zhao X. D. Han X. Chew J. L. Liu J. Chiu K. P. Choo A. (2007). Whole-genome mapping of histone H3 Lys4 and 27 trimethylations reveals distinct genomic compartments in human embryonic stem cells. *Cell Stem Cell* 1 286–298. 10.1016/j.stem.2007.08.004 18371363

[B68] Zhou V. W. Goren A. Bernstein B. E. (2011). Charting histone modifications and the functional organization of mammalian genomes. *Nat. Rev. Genet.* 12 7–18. 10.1038/nrg2905 21116306

