# Corrigendum: Putative Causal Variants Are Enriched in Annotated Functional Regions From Six Bovine Tissues

**DOI:** 10.3389/fgene.2021.757331

**Published:** 2021-09-21

**Authors:** Claire P. Prowse-Wilkins, Jianghui Wang, Ruidong Xiang, Josie B. Garner, Michael E. Goddard, Amanda J. Chamberlain

**Affiliations:** ^1^ Faculty of Veterinary and Agricultural Science, The University of Melbourne, Parkville, VIC, Australia; ^2^ Agriculture Victoria, AgriBio, Centre for AgriBiosciences, Bundoora, VIC, Australia; ^3^ Agriculture Victoria, Ellinbank Dairy Centre, Ellinbank, VIC, Australia

**Keywords:** bovine, ChIP-seq, Histone modifications, function, causal variants, differential binding, annotation, chromHMM

In the original article, there was an error in the formula described for calculation of enrichment. This was a mistake in the text only and was not the method used to calculate enrichment.

A correction has been made to **Materials and Methods, Enrichment of Putative Causal SNPs** in **Functional Regions, Paragraph 2:**

“Enrichment = (C/A)/(B/D) where: A is the number of positions under peaks, B is the number of positions that were putative causal SNPs, C is the number of positions under peaks and also a putative causal SNP and D is the number of positions in the genome”

The authors apologize for this error and state that this does not change the scientific conclusions of the article in any way. The original article has been updated.

