# Supplementary material for: Putative Causal Variants Are Enriched in Annotated Functional Regions From Six Bovine Tissues
Source: Front Genet. 2021 Jun 23;12:664379. doi: 10.3389/fgene.2021.664379 (PMC8260860; doi:10.3389/fgene.2021.664379)
Supplement: Supplementary Figure 1 — Pearson correlations of mapped reads with other parameters. (A). The number of peaks found significantly increases as mapped reads increases (r = 0.656, P < 0.001). (B). The percent of the genome covered by narrow peaks significantly increases as mapped reads increase (r = 0.715, P < 0.001). (C). The percent of the genome covered by broad peaks significantly increases as mapped reads increase (r = 0.363, P = 0.036). [file Image_1.PDF]

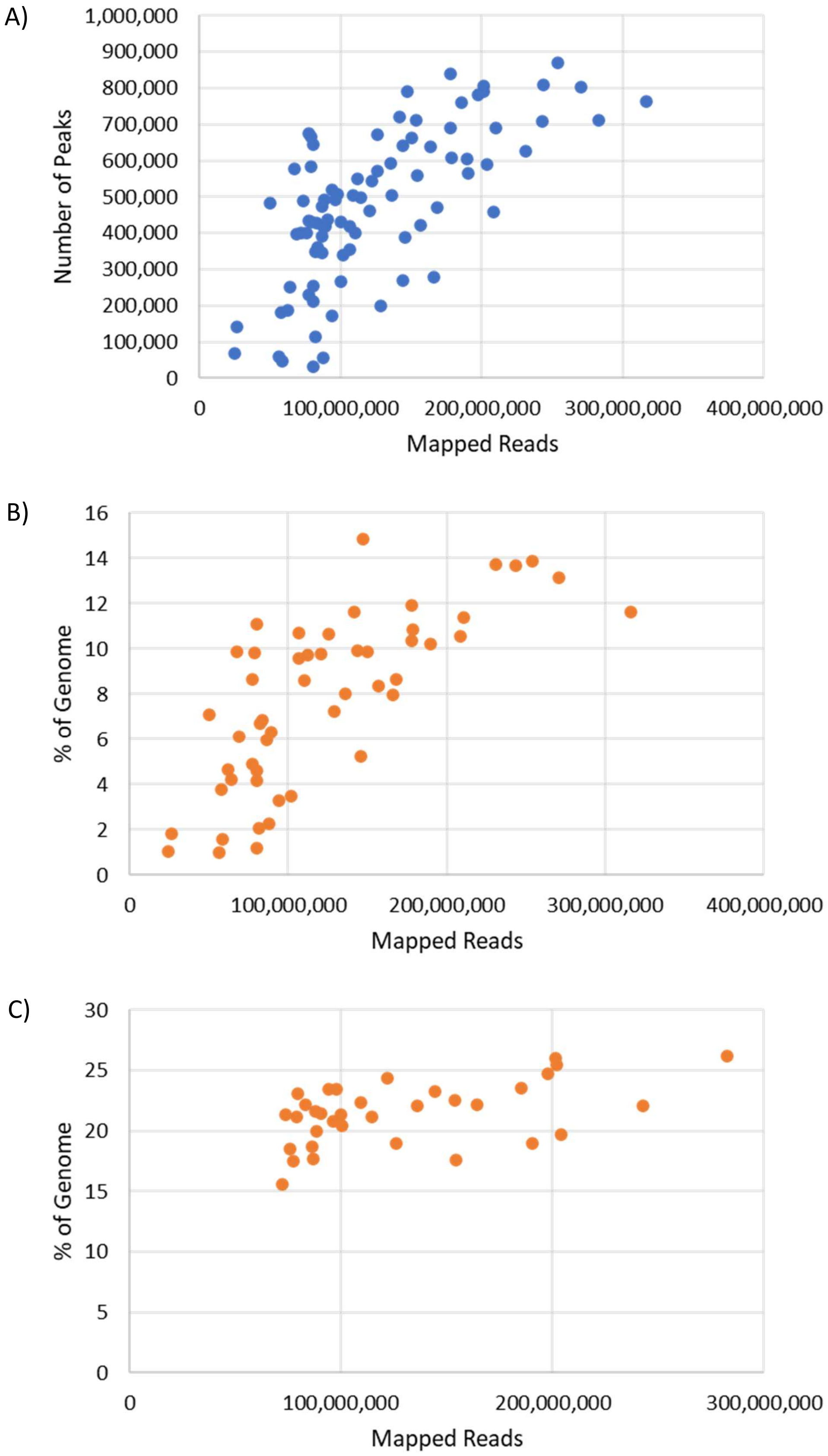

Supplementary Figure 1. Pearson correlations of mapped reads with other parameters. *A). The number of peaks found significantly increases as mapped reads increases (r=0.656, P<0.001). B). The percent of the genome covered by narrow peaks significantly increases as mapped reads increase (r=0.715, P<0.001). C). The percent of the genome covered by broad peaks significantly increases as mapped reads increase (r=0.363, P=0.036).*
