# Supplementary material for: Putative Causal Variants Are Enriched in Annotated Functional Regions From Six Bovine Tissues
Source: Front Genet. 2021 Jun 23;12:664379. doi: 10.3389/fgene.2021.664379 (PMC8260860; doi:10.3389/fgene.2021.664379)
Supplement: Supplementary Figure 2 — Comparison of profiles of H3K27Me3 and H3K27ac at active and inactive genes. Normalised ChIP-seq signal above input signal of the active (H3K27ac) and inactive (H3K27Me3) histone modifications within 2kb of transcription start sites (TSS) for active and inactive genes in all six tissues. [file Image_2.PDF]

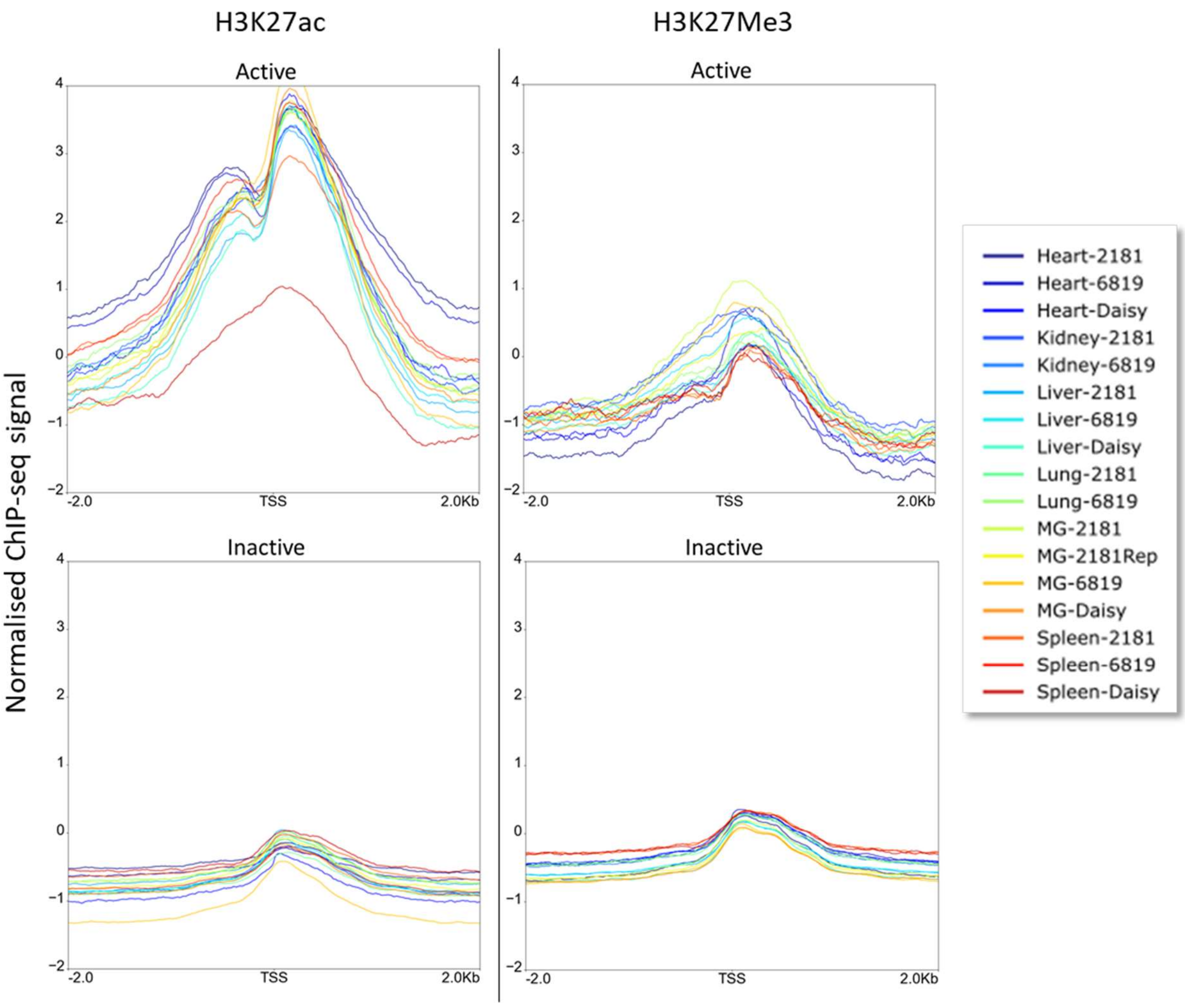

Supplementary Figure 2. Comparison of profiles of H3K27Me3 and H3K27ac at active and inactive genes*. Normalised ChIP-seq signal above input signal of the active (H3K27ac) and inactive (H3K27Me3) histone modifications within 2kb of transcription start sites (TSS) for active and inactive genes in all 6 tissues.*
