# Supplementary material for: Putative Causal Variants Are Enriched in Annotated Functional Regions From Six Bovine Tissues
Source: Front Genet. 2021 Jun 23;12:664379. doi: 10.3389/fgene.2021.664379 (PMC8260860; doi:10.3389/fgene.2021.664379)
Supplement: Supplementary Figure 3 — Comparison of replicates in each mark. Pearson correlations of replicates in each mark. [file Image_3.PDF]

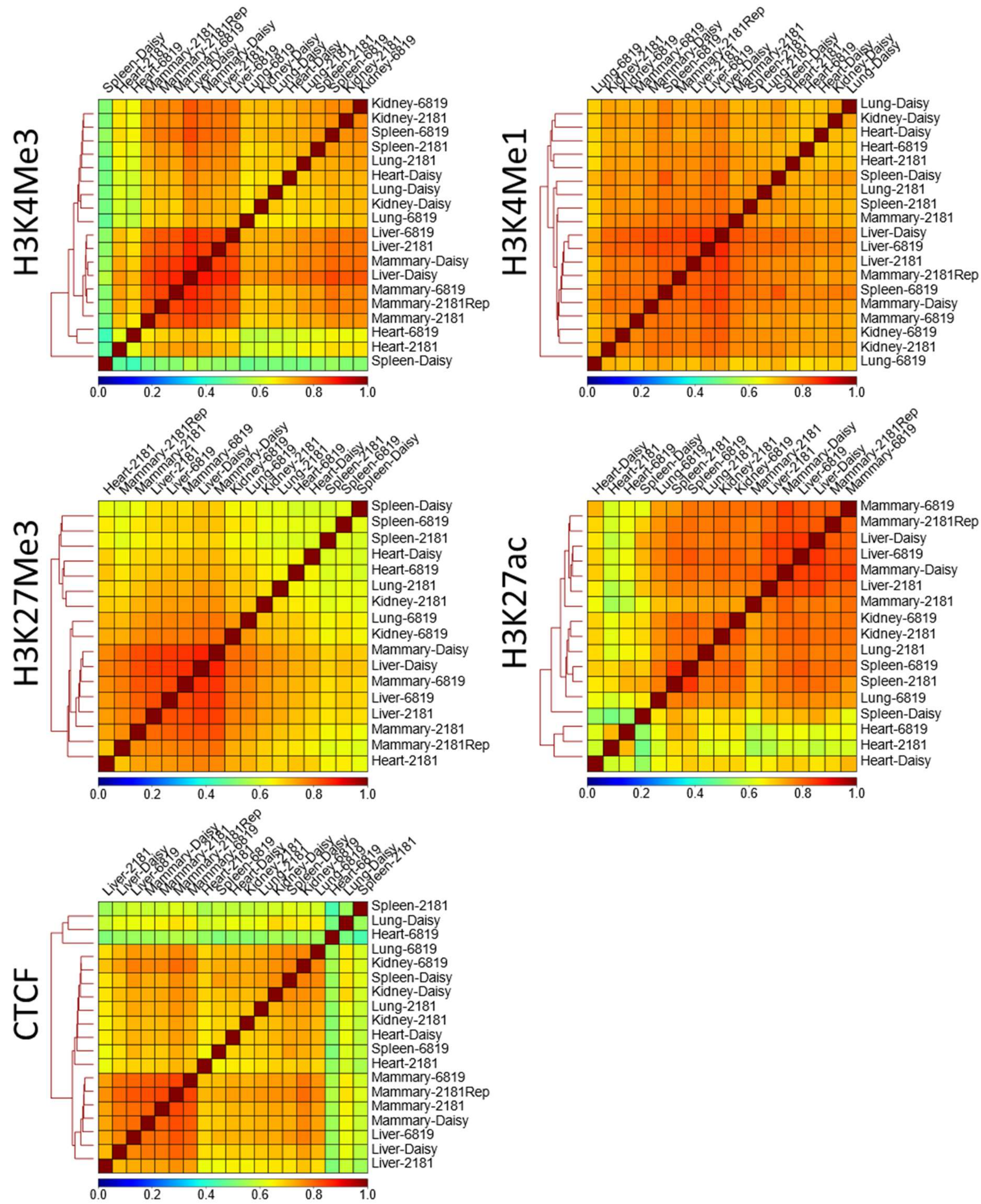


***Supplementary Figure 3. Comparison of replicates in each mark.*** *Pearson correlations of replicates in each mark.*
