# Supplementary material for: Putative Causal Variants Are Enriched in Annotated Functional Regions From Six Bovine Tissues
Source: Front Genet. 2021 Jun 23;12:664379. doi: 10.3389/fgene.2021.664379 (PMC8260860; doi:10.3389/fgene.2021.664379)
Supplement: Supplementary Figure 4 — Direction of differential binding. Number of differentially bound peaks with either higher (Up) or lower (Down) binding in heart, liver, kidney, lung, mammary gland (MG), and spleen. [file Image_4.PDF]

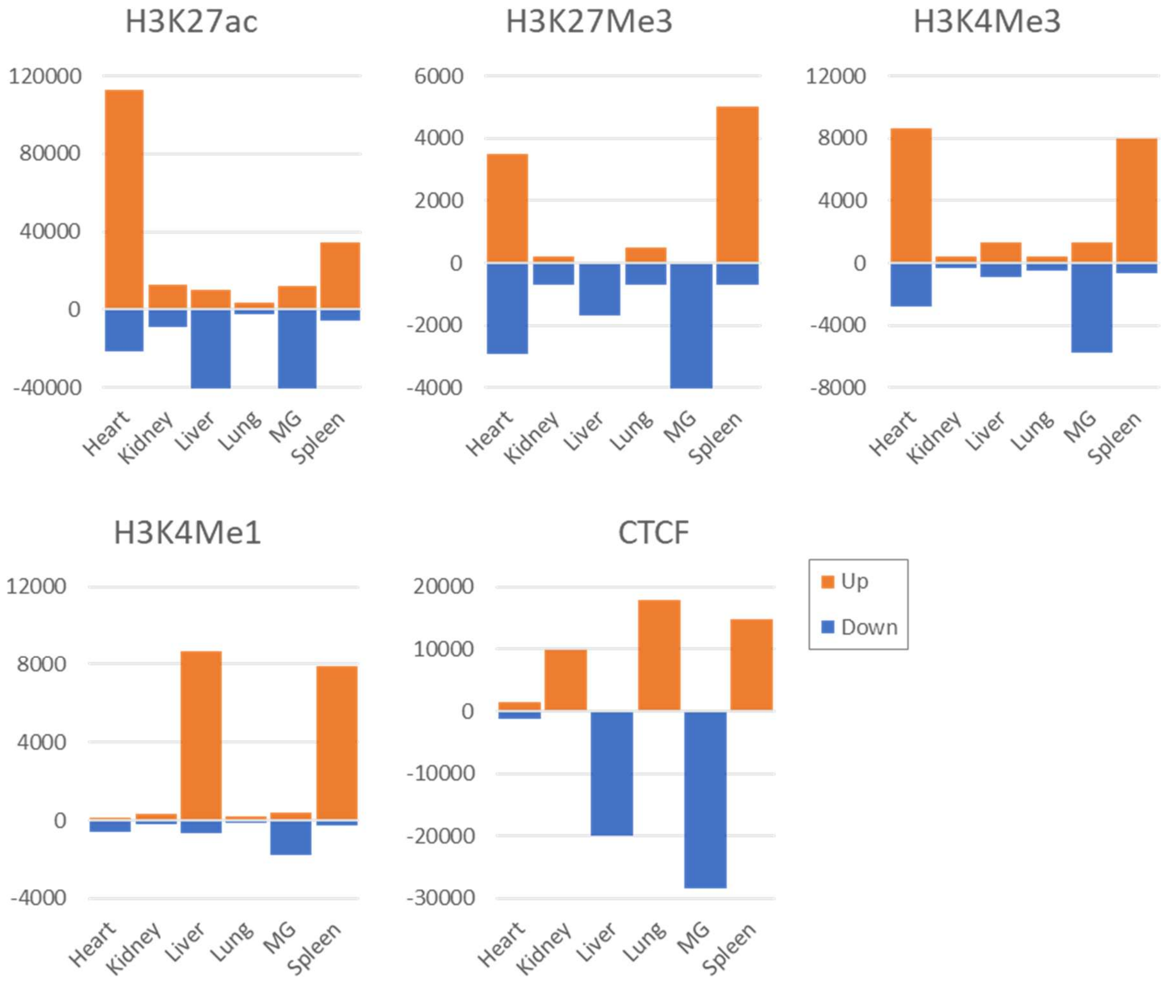

Supplementary Figure 4. Direction of differential binding. *Number of differentially bound peaks with either higher (Up) or lower (Down) binding in heart, liver, kidney, lung, mammary gland (MG) and spleen.*
