# Supplementary material for: Putative Causal Variants Are Enriched in Annotated Functional Regions From Six Bovine Tissues
Source: Front Genet. 2021 Jun 23;12:664379. doi: 10.3389/fgene.2021.664379 (PMC8260860; doi:10.3389/fgene.2021.664379)
Supplement: Supplementary Figure 5 — Enrichment of 7 Chromatin states in different tissues at annotated regions of the bovine genome. Where state1 is “inactive promoter,” state 2 is “repressed,” state 3 is “no signal,” state 4 is “hyperChIPable,” state 5 is “active enhancer,” state 6 is “permissive,” and state 7 is “active promoter.” Darker blue indicates higher enrichment. [file Image_5.PDF]

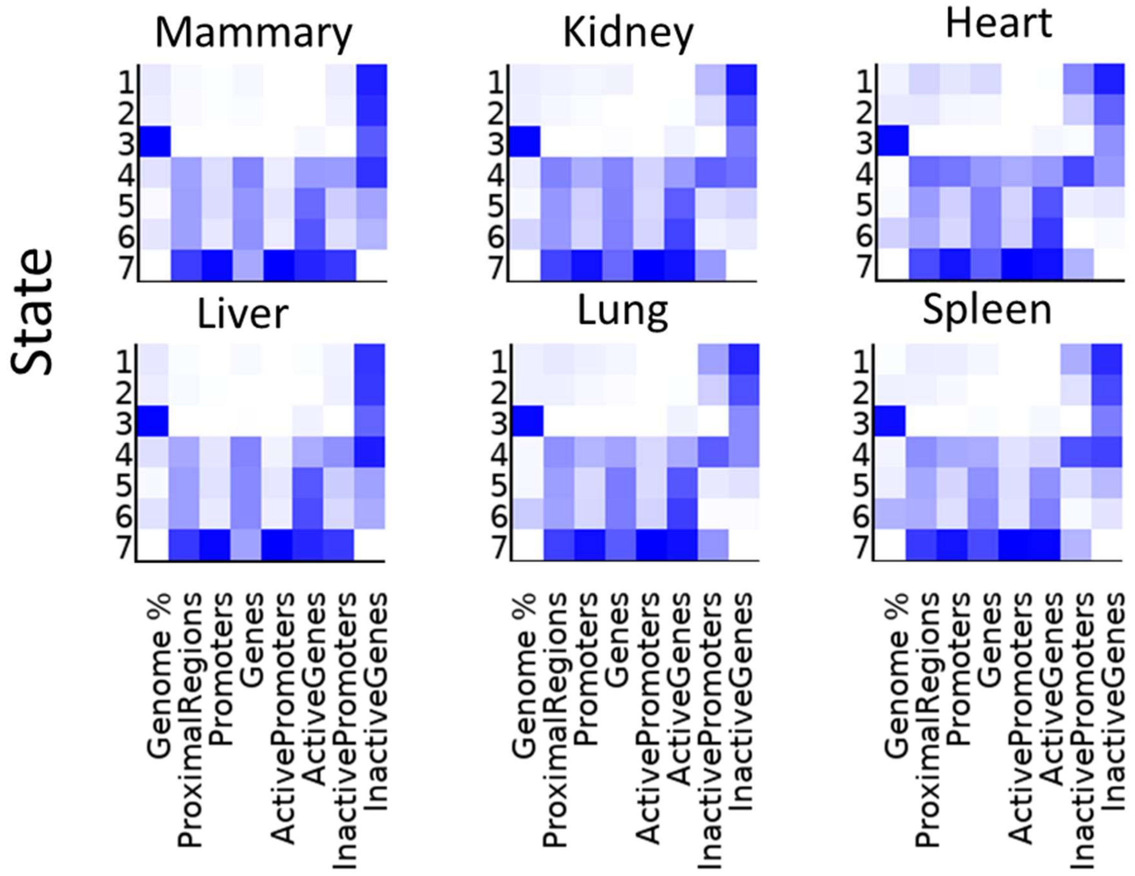

Supplementary Figure 5. Enrichment of 7 Chromatin states in different tissues at annotated regions of the bovine genome. *Where state1 is "inactive promoter", state 2 is "repressed", state 3 is "no signal", state 4 is "hyperChIPable", state 5 is "active enhancer", state 6 is "permissive" and state 7 is "active promoter". Darker blue indicates higher enrichment.*
