# Supplementary material for: Putative Causal Variants Are Enriched in Annotated Functional Regions From Six Bovine Tissues
Source: Front Genet. 2021 Jun 23;12:664379. doi: 10.3389/fgene.2021.664379 (PMC8260860; doi:10.3389/fgene.2021.664379)
Supplement: Supplementary Table 1 — Summary of 86 ChIP-seq datasets. The number of biological replicates is shown for each tissue-mark combination. [file Table_1.DOCX]

**Supplementary Table 1.** **Summary of 86 ChIP-seq datasets.** The number of biological replicates is shown for each tissue-mark combination.

|  | **H3K4Me1** | **H3K4Me3** | **H3K27Me3** | **H3K27ac** | **CTCF** |
| --- | --- | --- | --- | --- | --- |
| **Heart** | 3 | 3 | 3 | 3 | 3 |
| **Kidney** | 3 | 3 | 2 | 2 | 3 |
| **Liver** | 3 | 3 | 3 | 3 | 3 |
| **Lung** | 3 | 3 | 2 | 2 | 3 |
| **Mammary Gland** | 3 | 3 | 3 | 3 | 3 |
| **Spleen** | 3 | 3 | 3 | 3 | 3 |
