# Supplementary material for: Putative Causal Variants Are Enriched in Annotated Functional Regions From Six Bovine Tissues
Source: Front Genet. 2021 Jun 23;12:664379. doi: 10.3389/fgene.2021.664379 (PMC8260860; doi:10.3389/fgene.2021.664379)
Supplement: Supplementary Table 3 — Correlation between ChIP-seq and RNA-seq counts. The number of tests for correlation between gene expression and peak height as well as the number and percentage significantly correlated (P < 0.05) for each mark. All peaks within 100 kb of a gene were tested. [file Table_3.DOCX]

**Supplementary Table 3. Correlation between ChIP-seq and RNA-seq counts.** The number of tests for correlation between gene expression and peak height as well as the number and percentage significantly correlated (P<0.05) for each mark. All peaks within 100kb of a gene were tested.

| **Mark** | **Tests** | **Significantly correlated peak-gene pairs (percentage)** |
| --- | --- | --- |
| H3K27ac | 2,092,988 | 214,393 (10.2%) |
| H3K4Me1 | 975,002 | 108,379 (11.1%) |
| H3K4Me3 | 1,679,273 | 132,025 (7.9%) |
| H3K27Me3 | 1,388,674 | 98,901 (7.1%) |
| CTCF | 1,874,087 | 125,630 (6.7%) |
