# Supplementary material for: Putative Causal Variants Are Enriched in Annotated Functional Regions From Six Bovine Tissues
Source: Front Genet. 2021 Jun 23;12:664379. doi: 10.3389/fgene.2021.664379 (PMC8260860; doi:10.3389/fgene.2021.664379)
Supplement: Supplementary Table 4 — Active and inactive genes used for ChromHMM annotation. The number of genes defined as active, tissue-specific active and inactive based on normalised count data from RNA-seq in siz tissues across three animals. [file Table_4.DOCX]

**Supplementary Table 4. Active and inactive genes used for ChromHMM annotation.** The number of genes defined as active, tissue-specific active and inactive based on normalised count data from RNA-seq in 6 tissues across 3 animals.

| **Tissue** | **Active genes** | **Inactive genes** | **Tissue specific active genes** |
| --- | --- | --- | --- |
| Heart | 1826 | 11815 | 96 |
| Kidney | 1168 | 10653 | 14 |
| Liver | 1623 | 11283 | 134 |
| Lung | 1293 | 10804 | 11 |
| Mammary | 1354 | 11342 | 26 |
| Spleen | 1232 | 11187 | 6 |
