# Supplementary material for: Putative Causal Variants Are Enriched in Annotated Functional Regions From Six Bovine Tissues
Source: Front Genet. 2021 Jun 23;12:664379. doi: 10.3389/fgene.2021.664379 (PMC8260860; doi:10.3389/fgene.2021.664379)
Supplement: Supplementary Table 8 — Number of peaks correlated with gene expression. Number of peaks for each mark whose height correlated with gene expression. [file Table_8.DOCX]

**Supplementary Table 8. Number of peaks correlated with gene expression.** Number of peaks for each mark whose height correlated with gene expression.

| **Mark** | **Number of Peaks** |
| --- | --- |
| H3K4Me3 | 97,642 |
| H3K27ac | 148,039 |
| H3K4Me1 | 79,843 |
| H3K27Me3 | 72,344 |
| CTCF | 98,019 |
