# Supplementary material for: Putative Causal Variants Are Enriched in Annotated Functional Regions From Six Bovine Tissues
Source: Front Genet. 2021 Jun 23;12:664379. doi: 10.3389/fgene.2021.664379 (PMC8260860; doi:10.3389/fgene.2021.664379)
Supplement: Supplementary Table 9 — Enrichment of putative causal variants of peaks and peaks correlated with gene expression. Enrichment of each SNP dataset within peaks and peaks correlated with gene expression. Filtering for correlation with gene expression increased enrichment for causal variants. Enrichment was significant with P < 0.001 for all tests. [file Table_9.DOCX]

**Supplementary Table 9.** **Enrichment of putative causal variants of peaks and peaks correlated with gene expression.** Enrichment of each SNP dataset within peaks and peaks correlated with gene expression. Filtering for correlation with gene expression increased enrichment for causal variants. Enrichment was significant with P<0.001 for all tests.

|  | **H3K4Me3** | | **H3K27ac** | | **CTCF** | | **H3K4Me1** | | **H3K27Me3** | |
| --- | --- | --- | --- | --- | --- | --- | --- | --- | --- | --- |
|  | **Peaks** | **Correlated Peaks** | **Peaks** | **Correlated Peaks** | **Peaks** | **Correlated Peaks** | **Peaks** | **Correlated Peaks** | **Peaks** | **Correlated Peaks** |
| **Allele specific eQTL** | 1.86 | 2.6 | 1.96 | 2.34 | 1.93 | 2.46 | 1.76 | 2.21 | 1.69 | 2.27 |
| **Exon eQTL** | 1.68 | 2.26 | 2.21 | 2.01 | 1.73 | 2.19 | 1.61 | 1.99 | 1.33 | 2.02 |
| **Gene eQTL** | 2.24 | 3.77 | 2.37 | 3.14 | 2.27 | 3.58 | 1.97 | 2.94 | 1.82 | 2.99 |
| **Conserved regions** | 1.66 | 1.58 | 1.46 | 1.31 | 1.42 | 1.49 | 1.21 | 1.09 | 1.14 | 1.13 |
| **SNP 80k** | 1.2 | 1.08 | 1.16 | 1.11 | 1.18 | 1.07 | 1.16 | 1.08 | 1.15 | 1.08 |
| **Splice QTL** | 1.7 | 2.29 | 1.77 | 2.03 | 1.75 | 2.19 | 1.63 | 1.97 | 1.58 | 1.99 |
| **QTL Protein Yield** | 4.46 | 6.48 | 4.27 | 5.26 | 4.06 | 7.47 | 3.21 | 5.52 | 2.93 | 5.17 |
| **QTL Fat yield** | 3.72 | 4.03 | 3.46 | 3.3 | 3.43 | 5.46 | 2.82 | 3.66 | 2.6 | 3.84 |
| **QTL Milk Yield** | 3.09 | 3.32 | 2.79 | 2.63 | 2.85 | 4.45 | 2.35 | 2.77 | 2.24 | 2.77 |
| **QTL Fat percentage** | 2.78 | 3.12 | 2.51 | 2.42 | 2.58 | 3.6 | 2.19 | 2.27 | 2.16 | 2.27 |
| **QTL Protein percentage** | 1.85 | 2.75 | 1.91 | 2.22 | 1.8 | 3.26 | 1.58 | 2.4 | 1.4 | 2.45 |
