# Supplementary material for: Putative Causal Variants Are Enriched in Annotated Functional Regions From Six Bovine Tissues
Source: Front Genet. 2021 Jun 23;12:664379. doi: 10.3389/fgene.2021.664379 (PMC8260860; doi:10.3389/fgene.2021.664379)
Supplement: Supplementary Table 11 — The number of SNPs in each dataset grouped by distance to nearest transcription start site. For each SNP dataset we grouped SNPs based on distance to nearest transcription start site in bins of 100 kb. [file Table_11.DOCX]

**Supplementary Table 11. The number of SNP in each dataset grouped by distance to nearest transcription start site.** For each SNP dataset we grouped SNP based on distance to nearest transcription start site in bins of 100kb.

|  | **SNP 80k** | **Gene eQTL** | **Exon eQTL** | **Splice QTL** | **Conserved regions** | **Allele specific eQTL** | **QTL Protein yield** | **QTL Fat yield** | **QTL Milk yield** | **QTL Fat %** | **QTL Protein %** |
| --- | --- | --- | --- | --- | --- | --- | --- | --- | --- | --- | --- |
| **0-100kb** | 52233 | 101927 | 783599 | 924665 | 234630 | 944337 | 3158 | 4015 | 5369 | 8853 | 13331 |
| **100-200kb** | 13159 | 6092 | 99457 | 117116 | 63483 | 96972 | 86 | 720 | 1212 | 2228 | 2283 |
| **200-300kb** | 6568 | 1621 | 36014 | 40280 | 31946 | 34125 | 73 | 67 | 244 | 689 | 849 |
| **300-400kb** | 3592 | 472 | 13500 | 18676 | 18973 | 14326 | 0 | 1 | 20 | 292 | 447 |
| **400-500kb** | 2277 | 66 | 7475 | 7228 | 11000 | 6197 | 0 | 4 | 36 | 194 | 91 |
| **500-600kb** | 1279 | 12 | 3566 | 2600 | 6467 | 2730 | 0 | 0 | 0 | 67 | 8 |
| **600-700kb** | 839 | 10 | 1061 | 1284 | 3771 | 774 | 0 | 8 | 1 | 27 | 0 |
| **700-800kb** | 486 | 0 | 999 | 278 | 2488 | 802 | 0 | 0 | 1 | 19 | 0 |
| **800-900kb** | 355 | 0 | 80 | 167 | 1747 | 119 | 0 | 0 | 0 | 3 | 1 |
| **900kb-1mb** | 223 | 0 | 80 | 24 | 1115 | 48 | 0 | 0 | 0 | 0 | 1 |
