# Supplementary material for: Putative Causal Variants Are Enriched in Annotated Functional Regions From Six Bovine Tissues
Source: Front Genet. 2021 Jun 23;12:664379. doi: 10.3389/fgene.2021.664379 (PMC8260860; doi:10.3389/fgene.2021.664379)
Supplement: Supplementary Table 12 — The number of peaks grouped by distance to transcription start site, averaged over samples. The average number of peaks, grouped by distance to the nearest transcription start site, tested for enrichment. [file Table_12.DOCX]

**Supplementary Table 12. The number of peaks grouped by distance to transcription start site, averaged over samples.** The average number of peaks, grouped by distance to the nearest transcription start site, tested for enrichment.

|  | **H3K27ac** | **H3K4Me1** | **H3K4Me3** | **H3K27Me3** | **CTCF** |
| --- | --- | --- | --- | --- | --- |
| **0-100kb** | 364,580 | 343,633 | 293,124 | 358,911 | 328,395 |
| **100-200kb** | 57,466 | 75,006 | 54,554 | 87,284 | 57,566 |
| **200-300kb** | 21,509 | 32,633 | 23,286 | 40,785 | 24,038 |
| **300-400kb** | 9,793 | 16,530 | 11,565 | 21,827 | 11,825 |
| **400-500kb** | 5,379 | 9,527 | 6,696 | 12,928 | 6,829 |
| **500-600kb** | 2,865 | 5,414 | 3,799 | 7,687 | 3,865 |
| **600-700kb** | 1,488 | 3,065 | 2,116 | 4,557 | 2,131 |
| **700-800kb** | 896 | 1,887 | 1,308 | 2,863 | 1,317 |
| **800-900kb** | 612 | 1,291 | 904 | 1,967 | 914 |
| **900kb-1mb** | 375 | 839 | 574 | 1,312 | 587 |
