# Supplementary material for: Putative Causal Variants Are Enriched in Annotated Functional Regions From Six Bovine Tissues
Source: Front Genet. 2021 Jun 23;12:664379. doi: 10.3389/fgene.2021.664379 (PMC8260860; doi:10.3389/fgene.2021.664379)
Supplement: Supplementary Table 13 — Number of SNPs in milk production QTL datasets after filtering using r2. [file Table_13.DOCX]

**Supplementary Table 13. Number of SNP in GWAS milk production QTL datasets after filtering using r^2^.**

| **Dataset** | **Original Number of SNP** | **Filtered r^2^<0.5** |
| --- | --- | --- |
| QTL Protein Yield | 3,317 | 77 |
| QTL Fat yield | 4,815 | 144 |
| QTL Milk Yield | 6,883 | 169 |
| QTL Fat percentage | 12,373 | 376 |
| QTL Protein percentage | 17,012 | 317 |
